# USP13 facilitates pressure overload induced vascular remodeling and phenotypic transition of VSMCs via deubiquitinating Beclin-1

**DOI:** 10.1038/s41420-025-02931-w

**Published:** 2026-01-03

**Authors:** Rui-Qiang Qi, Qi-Fei Xie, Liu-Hang Su, Yan Wang, Sui-Ji Li, Xia Lu, Juan Song

**Affiliations:** 1 Xiamen Cardiovascular Hospital of Xiamen University, Xiamen University, Xiamen, Fujian China; 2 Department of Cardiology, Beijing Chaoyang Hospital, Capital Medical University, Beijing, China; 3 Department of Nuclear Medicine, The Third Affiliated Hospital of Soochow University, Changzhou, Jiangsu China; 4 Department of Cardiology, Shanghai Sixth People’s Hospital Affiliated to Shanghai Jiao Tong University School of Medicine, Shanghai, China

**Keywords:** Macroautophagy, Arterial stiffening, Renovascular hypertension, Ubiquitylation

## Abstract

Pressure overload-induced vascular remodeling is a complex physiological response that can result in detrimental cardiovascular diseases. Ubiquitination plays a critical role in this process; however, the role and specific mechanism of deubiquitinating enzyme USP13 in vascular remodeling remain poorly understood. Male C57BL/6J mice were subjected to pressure overload via transverse aortic constriction to investigate USP13’s effects in arterial remodeling. Primary vascular smooth muscle cells (VSMCs) were employed to investigate the role of USP13 on VSMC phenotype transition and potential mechanism. Mechanical stretch increased USP13 protein levels in vascular tissues while downregulating Acta2. Similarly, in both rat and human aortic VSMCs, PDGF-BB treatment significantly raised USP13 mRNA and protein levels. Notably, USP13 overexpression worsened arterial wall thickening in TAC mice and decreased Acta2 levels, whereas Spautin-1 treatment had a protective effect. At the cellular level, knocking down USP13 mitigated PDGF-BB-induced VSMC proliferation, as indicated by lower PCNA levels and reduced EdU (+) cell counts. Additionally, USP13 overexpression enhanced VSMC migration, demonstrated by scratch and transwell experiments. USP13 also aggravated PDGF-BB-induced downregulation of ACTA2 and Transgelin while promoting OST elevation. Mechanistically, USP13 interacted with Beclin-1, facilitating its deubiquitination and promoting autophagic flux, as shown by increased LC3 II/I ratios and decreased p62 levels. Moreover, BHLHE40 was explored as a new transcription factor of USP13, and BHLHE40 can regulate VSMCs proliferation and migration by transcriptionally activating USP13. In conclusion, our findings elucidate the role of USP13 in vascular remodeling under pressure overload, suggesting that targeting USP13 may offer therapeutic potential for pathological vascular disorders.

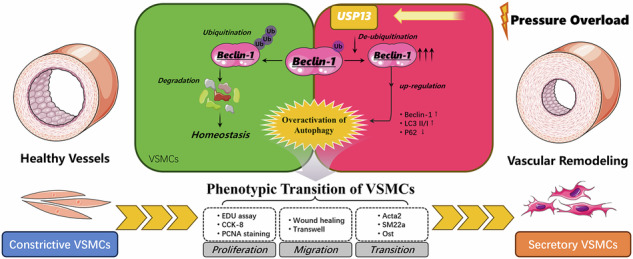

## Introduction

Vascular remodeling encompasses the structural and functional modifications that occur in blood vessels in response to pathological stimuli such as hypertension, atherosclerosis, and vascular injury [1]. This dynamic process involves significant alterations to the cellular composition and extracellular matrix of the vessel wall, leading to adverse outcomes such as increased vascular stiffness, lumen narrowing, and ultimately serious cardiovascular complications [1–3], including heart failure and myocardial infarction. As a result, vascular remodeling has emerged as a critical area of focus within cardiovascular research, as a comprehensive understanding of its underlying mechanisms is essential for developing therapeutic strategies aimed at alleviating the detrimental effects associated with these conditions. A key pathological feature of vascular remodeling is the phenotypic transition of vascular smooth muscle cells (VSMCs) [4, 5]. Under normal physiological conditions, VSMCs maintain a contractile phenotype characterized by elevated levels of contractile proteins and a quiescent state [6, 7]. However, in response to various stimuli, including growth factors and mechanical stress, VSMCs may undergo a phenotypic switch to a synthetic phenotype, marked by increased proliferation and enhanced production of extracellular matrix components [7]. This transition is crucial to the remodeling process, significantly contributing to arterial wall thickening and the formation of neointima, both of which are hallmarks of vascular disorders [5]. Despite the increasing understanding of these processes, the interplay between vascular remodeling and VSMC phenotypic transition remains complex and multifaceted.

Autophagy, an evolutionarily conserved lysosomal degradation pathway, plays a fundamental role in maintaining cellular homeostasis by recycling damaged organelles, misfolded proteins, and other cytoplasmic components [8]. This process involves the formation of double-membrane vesicles known as autophagosomes, which engulf cellular cargo and transport it to lysosomes for degradation. While basal autophagy is essential for cellular quality control and survival, dysregulated autophagy—whether through excessive activation or inhibition—has been implicated in the pathogenesis of various diseases, including cardiovascular disorders. In the cardiovascular system, autophagy serves as an adaptive response to stress signals such as oxidative stress, inflammation, and hemodynamic changes [9]. However, excessive autophagy in VSMCs has been identified as a key contributor to abnormal vascular remodeling.

Several stimuli—including platelet-derived growth factor-BB (PDGF-BB), angiotensin II (Ang-II), and mechanical stretch—have been shown to induce hyperactivation of autophagy in VSMCs, leading to phenotypic modulation, proliferation, migration, and extracellular matrix deposition—all hallmarks of vascular remodeling. For instance, PDGF-BB inhibits mTOR activity, thereby inducing autophagic flux in VSMCs, which contributes to hyperproliferation and neointima formation in a rat model of vascular injury [10]. Similarly, Ang-II, a key effector of the renin-angiotensin system, promotes hypertension and vascular inflammation by activating AT1 receptors on VSMCs. Ang-II has been shown to induce autophagy through NADPH oxidase-mediated reactive oxygen species (ROS) generation and endoplasmic reticulum (ER) stress. Ang-II treatment enhances the accumulation of LC3-II and promotes autophagosome formation in VSMCs, leading to increased cell migration and vascular fibrosis [11]. Mechanical stress, such as cyclic stretching resulting from hypertension or altered blood flow, also modulates autophagic activity in VSMCs. Increased mechanical stretch activates autophagy through integrin-mediated signaling and stretch-sensitive ion channels. These mechanisms are particularly relevant in hypertension-induced vascular hypertrophy and aneurysm formation. Despite its significance, the dual role of autophagy—as both a protective and detrimental process—complicates therapeutic targeting. Given its multifaceted functions, it is crucial to identify pivotal regulatory nodes to develop targeted therapies that can effectively modulate autophagy for the treatment of cardiovascular diseases.

Ubiquitination and de-ubiquitination have attracted considerable attention by regulating the stability of autophagy core molecules and thus influencing the progression of autophagic flow [12, 13]. Ubiquitin, a small regulatory protein, is covalently attached to substrate proteins, altering their stability, localization, and activity [14]. Meanwhile, deubiquitination, the removal of ubiquitin moieties from proteins, counterbalances ubiquitination and plays an equally important role in regulating protein function [15]. The ubiquitin-proteasome system (UPS) is the primary pathway through which ubiquitinated proteins are marked for degradation, a process predominantly mediated by E3 ubiquitin ligases [16]. In addition to E3 ligases, deubiquitinating enzymes (DUBs) play a pivotal role in reversing ubiquitination, thereby regulating the stability and activity of proteins [17]. The role of ubiquitination in cardiovascular disease has garnered increasing interest in recent years, particularly regarding its implications in vascular remodeling [18, 19]. Ubiquitin-specific peptidase 13 (USP13) is a member of the largest subfamily of cysteine protease deubiquitinating enzymes (DUBs) [20]. Previous studies have shown that USP13 plays a significant role in drug resistance and lung fibrosis by augmenting autophagy [21]. Importantly, research has demonstrated that autophagic activity critically regulates the phenotypic transition of VSMCs [11, 22], indicating that targeted modulation of autophagy may unveil novel therapeutic avenues for addressing vascular remodeling. However, it remains unclear whether USP13 contributes to phenotypic transitions and vascular remodeling induced by pressure overload.

In this study, we have, for the first time, investigated the role and mechanisms of USP13 in the phenotypic transition of VSMCs and the subsequent process of vascular remodeling. We found that protein levels of USP13 were significantly elevated in vascular tissues undergoing remodeling due to pressure overload. Additionally, in primary cultures of both rat and human VSMCs, we observed a time-dependent increase in USP13 protein levels in response to stimulation with platelet-derived growth factor-BB (PDGF-BB). Further in vivo experiments indicated that overexpression of USP13 resulted in an increase in vascular wall thickness and a concomitant downregulation of the contractile protein ACTA2. In contrast, intervention with USP13 inhibitors, Spautin-1, demonstrated therapeutic effects on vascular remodeling. Moreover, in vitro studies revealed that USP13 overexpression enhanced migration capacity, as assessed by wound healing and transwell migration assays. CCK-8 assays and EdU staining further indicated that USP13 overexpression promoted VSMC proliferation. Mechanistically, co-immunoprecipitation (Co-IP) and fluorescence co-localization assays revealed an interaction between USP13 and Beclin-1, suggesting that USP13 plays a role in the modulation of autophagy. Notably, we observed that USP13 deubiquitinates Beclin-1, promoting autophagic flux through the upregulation of the LC3B-II/I ratio and Beclin-1 expression while downregulating p62 protein levels. Interestingly, using siRNA to knock down Beclin-1, we demonstrated that this manipulation attenuates the adverse impact of USP13 overexpression on VSMCs migration, as observed in wound healing and Transwell migration assays. Similarly, EdU and PCNA staining experiments revealed that Beclin-1 depletion mitigates USP13 induced enhancement of VSMCs proliferation. In summary, our study elucidates the role of USP13 in enhancing autophagic flow and contributing to vascular remodeling through the deubiquitination of Beclin-1. These findings highlight potential therapeutic targets within the USP13-Beclin-1-Autophagy axis for the treatment of pressure overload-induced vascular remodeling.

## Results

### USP13 expression is upregulated in VSMCs differentiation and vascular remodeling

**Fig. 1:**
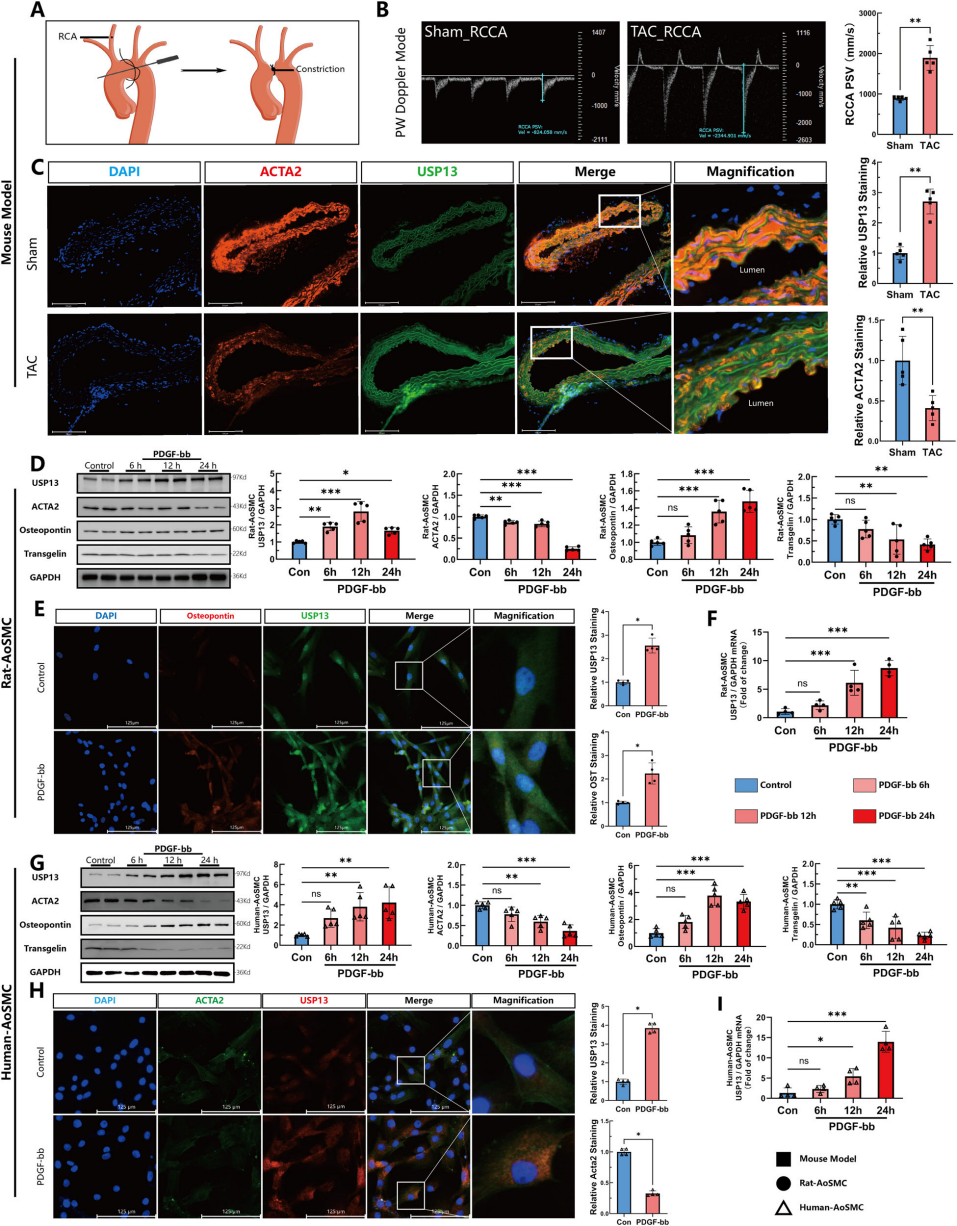
The expression of USP13 is up-regulated in pressure overload induced vascular remodeling and PDGF-BB induced phenotypic transition of VSMCs. **A** Surgical model drawing of transverse aortic construction (TAC). **B** Representative ultrasound doppler images and quantifications of right carotid artery blood flow in Sham and TAC groups (*n* = 5). **C** Immunofluorescence staining analysis for protein levels of Acta2 (Red), USP13 (Green), and DAPI (Blue) in Sham and TAC model (*n* = 5). Scale bars = 125 μm. **D** Representative western blot images and quantifications of USP13, Acta2, Osteopontin and Transgelin in Rat-AoSMCs treated with Control or PDGF-BB. (*n* = 5). **E** Immunostaining analysis of USP13 (Green) and Osteopontin (Red) for Rat-AoSMCs treated with Control or PDGF-BB (*n* = 4). Scale bars = 125 μm. **F** Quantitative reverse transcription PCR (qRT-PCR) analysis of USP13 in Rat-AoSMCs (*n* = 4). **G** Representative western blot images and quantifications of USP13, Acta2, Osteopontin and Transgelin in Hu-AoSMCs treated with Control or PDGF-BB (*n* = 5). **H** Immunostaining analysis of USP13 (Red) and Acta2 (Green) for Hu-AoSMCs treated with Control or PDGF-BB (*n* = 4). Scale bars = 125 μm. **I** Quantitative reverse transcription PCR (qRT-PCR) analysis of USP13 in Hu-AoSMCs (*n* = 4). RCA right common carotid artery, PSV Peak Systolic Velocity, TAC transverse aortic construction, PDGF platelet-derived growth factor, Ao-SMCs Aortic vascular smooth muscle cells, DAPI 4’,6-diamidino-2-phenylindole. *p < 0.05, **p < 0.01, ***p < 0.001.

To investigate the relationship between USP13 and vascular remodeling, we first established a mouse model of vascular remodeling through transverse aortic constriction (TAC) surgery, a well-established experimental method to increase pressure in the right carotid artery (Fig. 1A). Ultrasound imaging of the right carotid artery revealed a significant post-operative increase in blood flow, confirming the successful induction of a pressure overload model for vascular remodeling (Fig. 1B). Subsequently, we observed an elevation in the protein levels of USP13 alongside a decrease in the expression of the vascular smooth muscle contractile marker ACTA2, as demonstrated through vascular tissue immunofluorescence (Fig. 1C). To further elucidate these findings in vitro, we isolated primary vascular smooth muscle cells from rat’s aorta and identified through immunofluorescence staining (Fig. S1). Following this, we stimulated the Rat VSMCs with PDGF-BB to promote VSMCs differentiation. Notably, Western blot and rt-qPCR analysis revealed that the protein and mRNA levels of USP13 progressively increased at 6, 12, and 24 h post-PDGF-BB treatment (Fig. 1D). This was accompanied by a downregulation of contractile proteins, including ACTA2 and Trangelin, alongside an upregulation of OST. Consistent with these observations, human aortic VSMCs also exhibited a time-dependent increase in USP13 protein levels upon PDGF-BB stimulation, further supporting our hypothesis (Fig. 1G, H). Immunofluorescence results mirrored these trends, revealing an upregulation of USP13 proteins in conjunction with a downregulation of Acta2 (Fig. 1H). Overall, these data collectively suggest a relationship between USP13 and vascular remodeling, suggesting its potential role in modulating VSMC differentiation and function in response to PDGF-bb.

### Expression of USP13 and its role in VSMCs function under pathological mechanical stretch

**Fig. 2:**
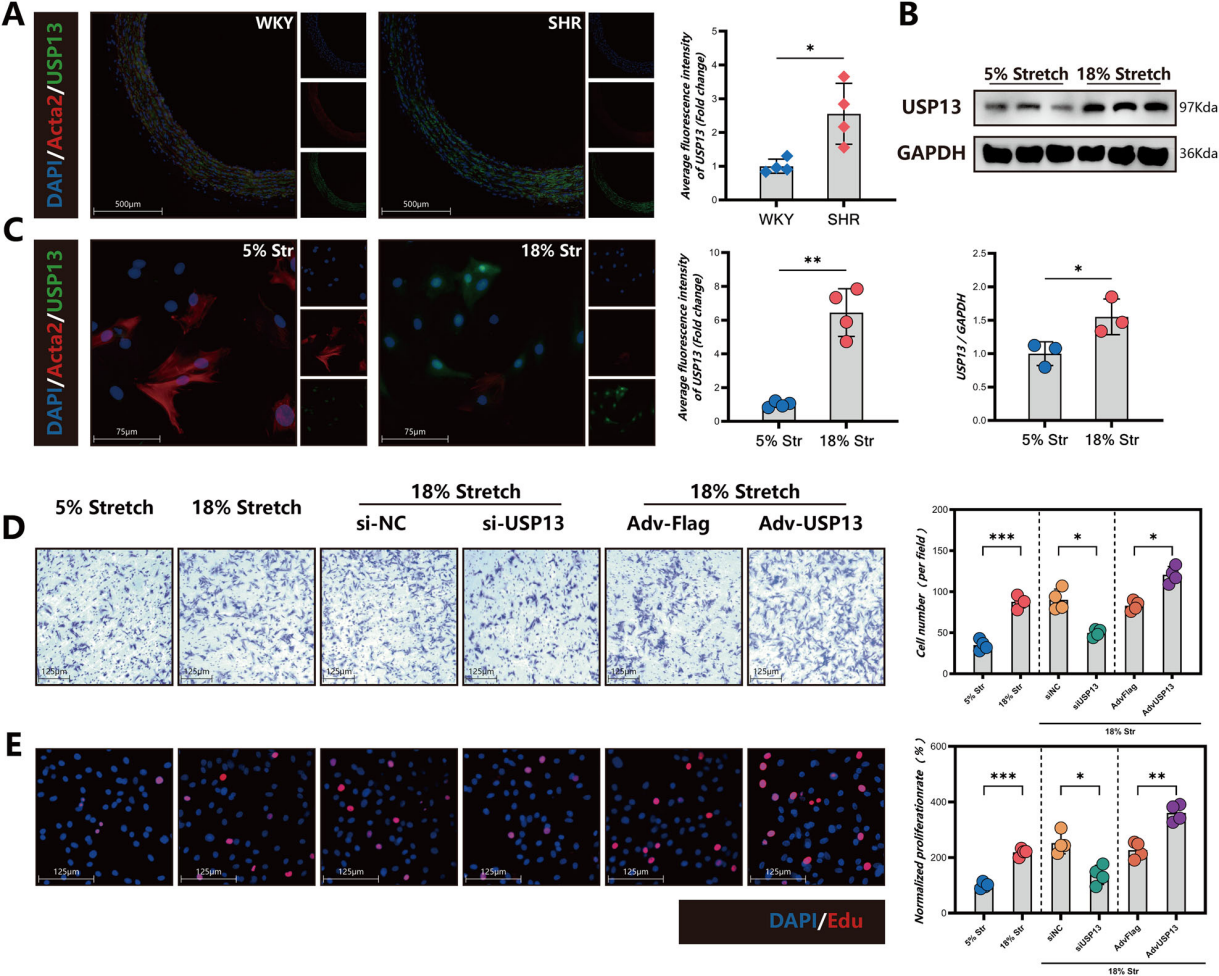
Expression of USP13 and its role in VSMCs function under pathological mechanical stretch. **A** Immunofluorescence staining analysis for protein levels of Acta2 (Red), USP13 (Green), and DAPI (Blue) in WKY and SHR model (*n* = 4). Scale bars = 500 μm. **B** Representative western blot images and quantifications of USP13 in primary Rat VSMCs treated with 5% or 18% stretch. **C** Immunofluorescence staining analysis for protein levels of Acta2 (Red), USP13 (Green), and DAPI (Blue) in primary Rat VSMCs treated with 5% or 18% stretch. (*n* = 4). Scale bars = 75 μm. **D** Representative images and quantitative analysis of the Transwell assay for VSMCs (*n* = 4, scale bars = 125 μm). **E** Representative EdU staining images and corresponding quantitative analysis (*n* = 4, scale bars = 125 μm). *p < 0.05, **p < 0.01, ***p < 0.001.

Next, we investigated the expression changes of USP13 in a hypertensive rat model. Immunofluorescence analysis revealed that the expression level of USP13 in the aorta of Spontaneously Hypertensive Rats was significantly higher than that observed in the Wistar Kyoto control group (Fig. 2A). Mechanical stretching serves as a critical pathological stimulus for VSMCs under conditions of pressure overload. When VSMCs were subjected to 18% mechanical stretch, we observed a substantial increase in USP13 protein levels compared to those subjected to 5% physiological stretch, as confirmed by both immunofluorescence and Western blot analyses (Fig. 2B, C). Furthermore, we examined the impact of USP13 intervention on VSMC function. Results from Transwell migration assays (Fig. 2D) and EdU staining (Fig. 2E) indicated that knockdown of USP13 significantly inhibited the heightened proliferation and migratory capacities of VSMCs induced by pathological mechanical stretch. Conversely, overexpression of USP13 yielded opposing effects, enhancing both proliferation and migration abilities. In summary, our findings underscore the significant upregulation of USP13 in hypertensive conditions and its important role in modulating VSMC function under mechanical stress.

### Targeting USP13 in vivo ameliorates pressure overload-induced vascular remodeling

**Fig. 3:**
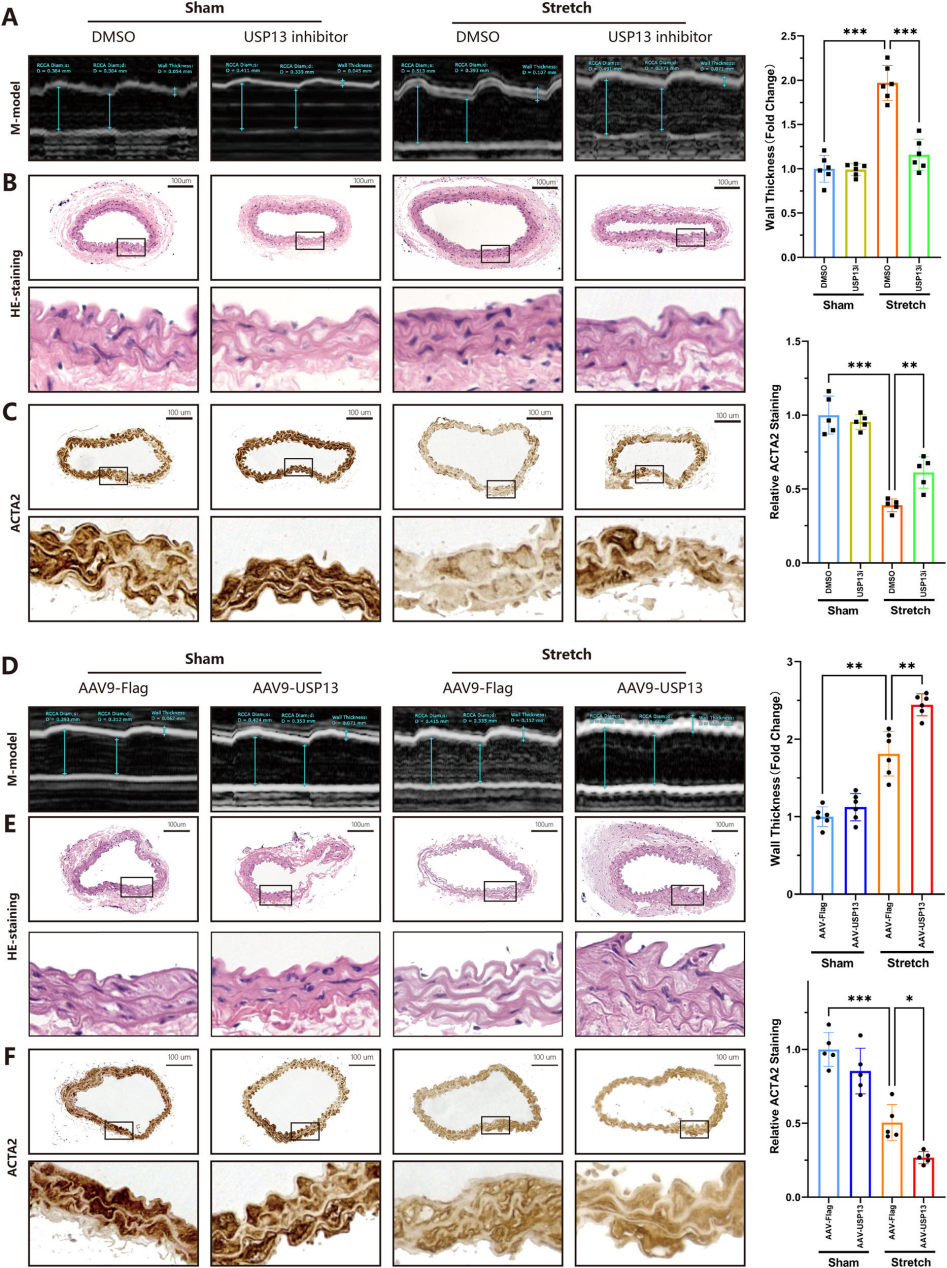
Targeting USP13 in-vivo ameliorates pressure overload-induced vascular remodeling. Spautin-1 was used to inhibit USP13 in vivo. After 4 weeks of intervention with Spautin-1, the mice were euthanized, and the carotid artery were harvested for subsequent analyses. **A** Representative M-mode images of right common carotid artery and quantifications of wall thickness (*n* = 6). **B** Representative hematoxylin-eosin (HE) staining of aortic sections, scale bars = 100 μm. **C** Representative immunohistochemical staining images and quantifications of Acta2 (*n* = 5, scale bars = 100 μm). Adeno-associated virus serotype 9 was utilized for the in vivo overexpression of USP13. **D** Representative M-mode images of the right common carotid artery and quantifications of wall thickness (*n* = 6 per group). **E** Representative hematoxylin-eosin (HE) staining of aortic sections, scale bars = 100 μm. **F** Representative immunohistochemical staining images and quantifications of Acta2 (*n* = 5, scale bars = 100 μm). *p < 0.05, **p < 0.01, ***p < 0.001.

To elucidate the role of USP13 in vascular remodeling, TAC induced vascular remodeling was conducted in-vivo experiments. As demonstrated in Fig. 3A, ultrasound M-mode imaging of the mouse carotid artery showed that the administration of an USP13 inhibitor effectively alleviated the increase in vascular wall thickness induced by pressure overload. This observation was further supported by similar patterns noted in HE-staining (Fig. 3B). Concurrently, ACTA2 immunohistochemical staining indicated that Spautin-1 could prevent the pressure overload-induced reduction of ACTA2 protein levels (Fig. 3C). We then investigated the effects of USP13 overexpression using AAV9-mediated delivery, confirmed via immunofluorescence (Fig. S2). As expected, USP13 overexpression exacerbated the thickening of the vascular wall and the decline in ACTA2 levels under pressure overload conditions (Fig. 3D). Interestingly, neither overexpression nor inhibition of USP13 significantly affected vascular wall thickness or ACTA2 protein levels under normal physiological conditions, suggesting a pivotal regulatory role for USP13 specifically in pathological scenarios (Fig. 3F). In summary, our findings underscore the influence of USP13 on vascular remodeling, highlighting its potential as a therapeutic target in pressure overload states.

### Knock down USP13 alleviates PDGF-BB induced phenotypic transition of VSMCs

**Fig. 4:**
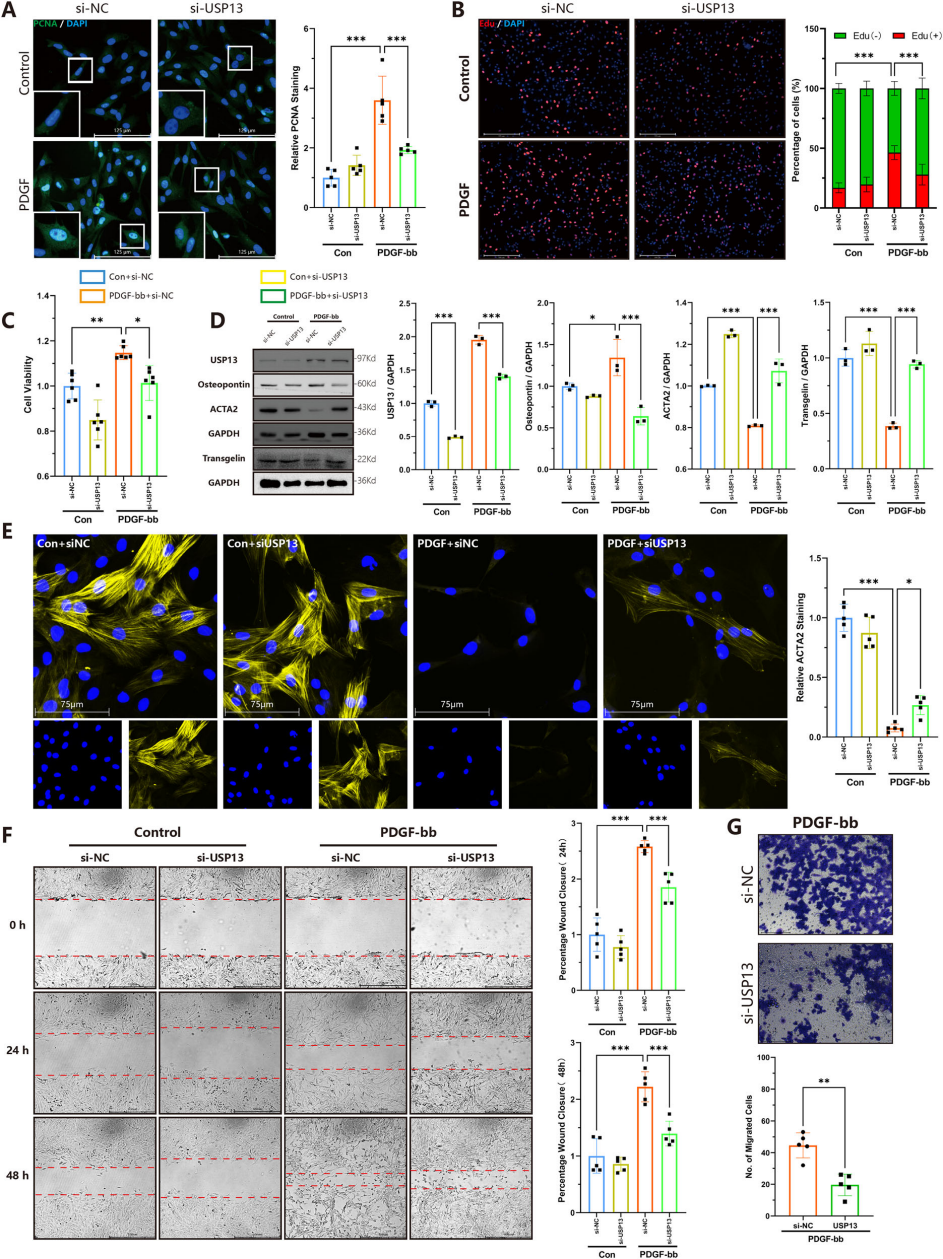
Knock down of USP13 mitigates phenotypic transition of rat VSMC induced by PDGF-BB. Small interfering RNAs were used to knock down USP13 expression in Rat-SMC under PDGF-bb stimulation. **A** Immunostaining analysis of PCNA (Green) and DAPI (Blue) accompanied by quantitative analysis (*n* = 5, scale bars = 125 μm). **B** Representative EdU staining images and corresponding quantitative analysis (*n* = 5, scale bars = 275 μm). **C** The CCK-8 method was performed to detect the cell viability of VSMCs (*n* = 6). **D** Representative Western blot images and quantitative analysis of USP13, Osteopontin, Acta2 and Transgelin protein levels (*n* = 3). **E** Immunostaining analysis of Acta2 (Yellow) and DAPI (Blue) accompanied by quantitative analysis (*n* = 5, scale bars = 125 μm). **F** Representative images and quantitative analysis of the wound healing assay for VSMCs (*n* = 5, scale bars = 650 μm). **G** Representative images and quantitative analysis of the Transwell assay for VSMCs (*n* = 5, scale bars = 650 μm). *p < 0.05, **p < 0.01, ***p < 0.001.

Considering the upregulation of USP13 expressions in vascular remodeling and the protective role of USP13 inhibitor, we hypothesized that USP13 might regulate this process by influence phenotypic transition of VSMCs. High proliferation and migration abilities are two key characteristics of VSMCs differentiation. We utilized siRNA to knock down USP13 in VSMCs, verifying the interference effect with Western Blot analysis (Fig. S3). Immunofluorescence results for PCNA, a marker for cell proliferation, indicated that USP13 knockdown inhibited the PDGF-bb induced increase in PCNA protein levels (Fig. 4A). Additionally, EdU staining (Fig. 4B) demonstrated that reduced USP13 levels decreased the number of EdU-positive cells, while CCK8 assays (Fig. 4C) confirmed a reduction in overall VSMCs numbers. On a molecular level, Western Blot analysis showed that USP13 knockdown impeded the PDGF-bb induced downregulation of ACTA2 and Transgelin, as well as the upregulation of OST (Fig. 4D). Subsequently, we assessed the migration capacity of VSMCs through wound healing (Fig. 4F) and Transwell assays (Fig. 4G). Our data revealed that USP13 knockdown significantly inhibited the PDGF-bb induced migration of VSMCs, as evidenced by reduced wound closure in scratch assays. Correspondingly, a decrease in the number of migrating cells was observed in the Transwell assays upon USP13 knockdown. In summary, our findings collectively indicate that the knockdown of USP13 effectively mitigates the enhanced proliferation and migration capabilities of VSMCs induced by PDGF-bb stimulation.

### Overexpression USP13 exacerbates phenotypic transition of VSMCs

**Fig. 5:**
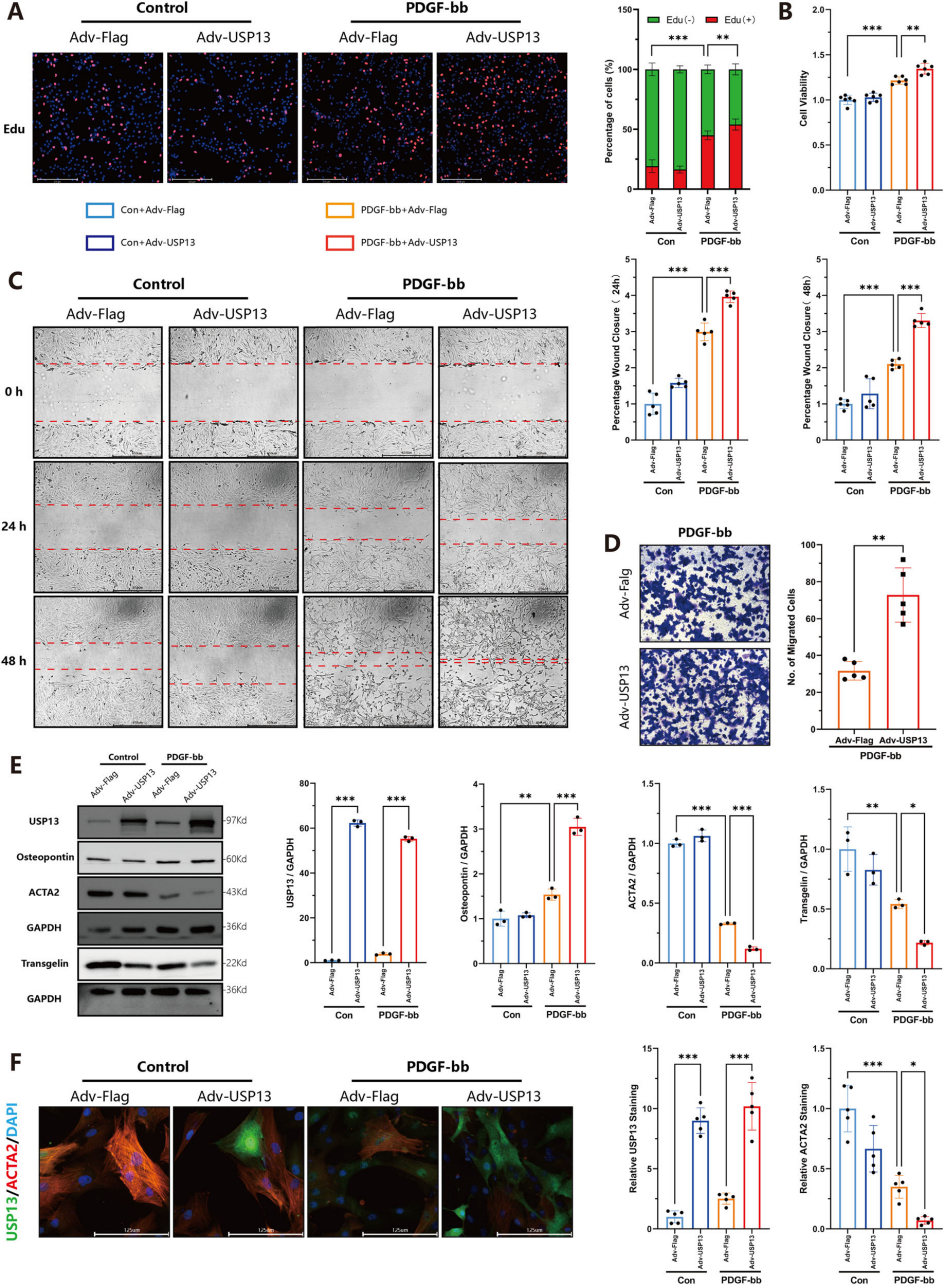
Overexpression of USP13 via adenovirus enhances the proliferation and migratory capacity of VSMCs and exacerbates VSMCs differentiation in vitro. **A** Representative EdU staining images and corresponding quantitative analysis (*n* = 5, scale bars = 275 μm). **B** The CCK-8 method was performed to detect the cell viability of VSMCs (*n* = 6). **C** Representative images and quantitative analysis of the wound healing assay for VSMCs (*n* = 5, scale bars = 650 μm). **D** Representative images and quantitative analysis of the Transwell assay for VSMCs (*n* = 5, scale bars = 650 μm). **E** Representative Western blot images and quantitative analysis of USP13, Osteopontin, Acta2 and Transgelin protein levels (*n* = 3). **F** Immunostaining analysis of Acta2 (Red), USP13 (Green) and DAPI (Blue) accompanied by quantitative analysis (*n* = 5, scale bars = 125 μm). *p < 0.05, **p < 0.01, ***p < 0.001.

To further elucidate the role of USP13 in VSMCs, we overexpressed USP13 in vitro using adenovirus and confirmed the overexpression via Western Blot analysis (Fig. S4). As anticipated, the overexpression of USP13 led to enhanced proliferation and migration capabilities in the VSMCs. As illustrated in Fig. 5, USP13 overexpression resulted in a modest increase in the number of EdU-positive VSMCs (Fig. 5A), and the CCK8 assay (Fig. 5B) corroborated that USP13 exacerbated the PDGF-bb-induced increase in VSMC numbers. The scratch assay (Fig. 5C) also demonstrated that USP13 overexpression significantly reduced the wound area at both 24- and 48-h post-incubation, while the Transwell assay (Fig. 5D) further corroborated the promotion of cell migration. Similar results were also observed in vascular smooth muscle cells derived from mice (Fig. S8). The Western Blot findings were consistent with immunofluorescence results, indicating that USP13 overexpression facilitated the PDGF-bb-induced downregulation of contractile markers Acta2 and Transgelin, alongside the upregulation of the synthetic marker OST (Fig. 5E). In conclusion, these results affirm the pivotal role of USP13 in modulating VSMC behavior, particularly in enhancing their proliferative and migratory responses to PDGF-bb stimulation.

### USP13 directly interacts with Beclin-1 and regulates de-ubiquitination of Beclin-1

**Fig. 6:**
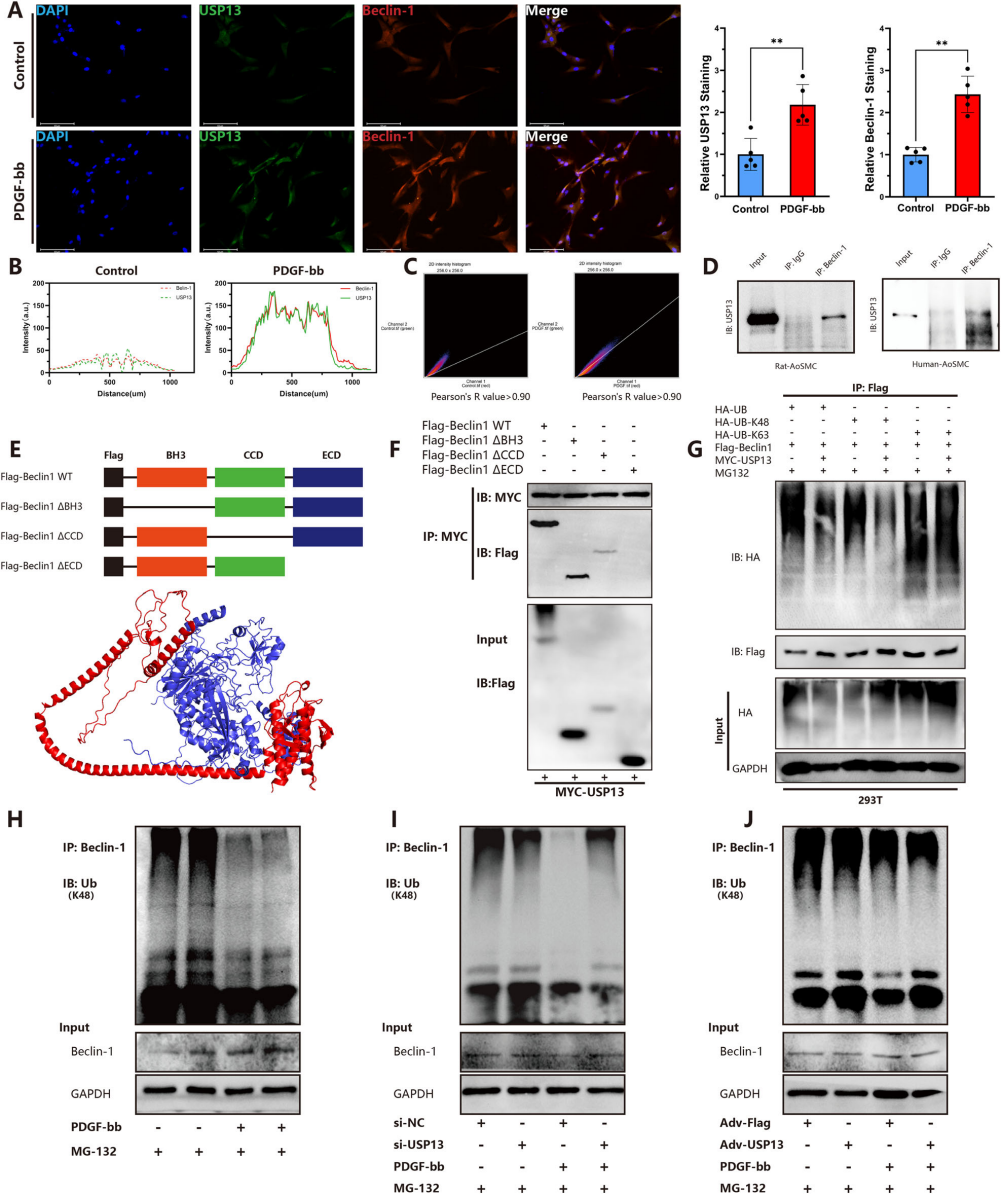
USP13 interacts and regulates the de-ubiquitination with Beclin-1. **A** Immunostaining of USP13 (Green) and Beclin-1 (Red) accompanied by quantitative analysis in rat-AoSMCs (*n* = 5, scale bars = 125 μm). **B**, **C** Co-localization and correlation analysis of USP13 and Beclin-1 in rat-AoSMCs. **D** Anti-Beclin-1 IP followed by Western blot in VSMCs. Anti- IgG IP was used as a negative control in Rat-AoSMCs and Human-AoSMCs. **E** The illustration of BECN1 deletion mutants and schematic representation of the binding interaction between USP13 and Beclin-1. **F** Exogenous normal or mutated Beclin-1 was immunoprecipitated by anti-Flag antibody. **G** Western blot analysis of indicated proteins in 293 T cells co-transfected with Flag-Beclin-1 and HA-Ub, HA-K48 and HA-K63 in the presence of Flag-vector or Flag-USP13 plus the proteasome inhibitor MG132 (10 μM) for 4 h before IP of whole cell lysates with Flag magnetic beads. **H** Rat-AoSMCs were treated with PDGF-bb and then subjected to MG132 (10 μM). Ubiquitinated Beclin-1 was detected by immunoblotting. The lysates were immunoprecipitated with anti-Beclin-1 antibody and immunoblotted with anti-ubiquitin (K48) antibody. **I**, **J** The ubiquitination level of Beclin-1 was evaluated through ubiquitination assays, after overexpressing or knocking down USP13. **p < 0.01.

Fluorescence co-localization studies indicated a significant overlap between USP13 and Beclin-1 fluorescence signals both under physiological conditions and after PDGF-bb stimulation, with Pearson’s correlation coefficients exceeding 0.9, suggesting a potential interaction between the two proteins (Fig. 6A–C). To substantiate these findings, co-immunoprecipitation (Co-IP) assays were performed, demonstrating that the anti-Beclin1 antibody effectively precipitated USP13, confirming the specificity of this interaction in both human and rat VSMCs (Fig. 6D). Subsequently, to identify the binding site of USP13 on Beclin-1, we employed cells expressing one of three BECN1 deletion mutants alongside MYC–USP13 constructs for Co-IP assays. Moreover, wild-type plasmids as well as truncated mutants of Beclin-1 were generated, targeting key functional domains: BCL2 homolog3 (BH3) domain, coiled-coil (CCD) domain, and the evolutionarily conserved domain (ECD) (Fig. 6E). Co-IP assays revealed that deletion of the ECD specifically disrupted the binding between USP13 and Beclin-1, indicating the essential role of the ECD in this interaction (Fig. 6F).

To further investigate the deubiquitination capabilities of USP13, we transfected 293 T cells with HA-ub, HA-ub-K48, and HA-ub-K63 plasmids to explore the ubiquitination modification sites of Beclin-1 (Fig. 6G). Ubiquitination assays established that USP13 is capable of deubiquitinating both full-length ubiquitin and K48-linked ubiquitin, suggesting that USP13 plays a significant role in preventing the degradation of Beclin-1 by removing K48-linked polyubiquitin chains, thereby stabilizing Beclin-1 and facilitating its function in autophagy. In primary VSMCs (Fig. 6H–J), the role of USP13 in the deubiquitination of Beclin-1 was further investigated. Treatment with PDGF-bb in conjunction with the proteasome inhibitor MG132 resulted in a significant reduction in K48-linked ubiquitination of Beclin-1, leading us to hypothesize that USP13 regulates Beclin-1 through deubiquitination. This hypothesis was confirmed through overexpression and knockdown experiments of USP13 in VSMCs. As expected, USP13 knockdown using siRNA abrogated the PDGF-bb induced reduction of Beclin-1 K48 ubiquitination (Fig. 6I), whereas adenoviral-mediated overexpression of USP13 yielded the opposite effect (Fig. 6J). Collectively, these results elucidate the crucial regulatory role of USP13 in modulating the ubiquitination and expression of Beclin-1 in response to PDGF-bb stimulation.

### USP13 regulates the autophagy activity of VSMCs

**Fig. 7:**
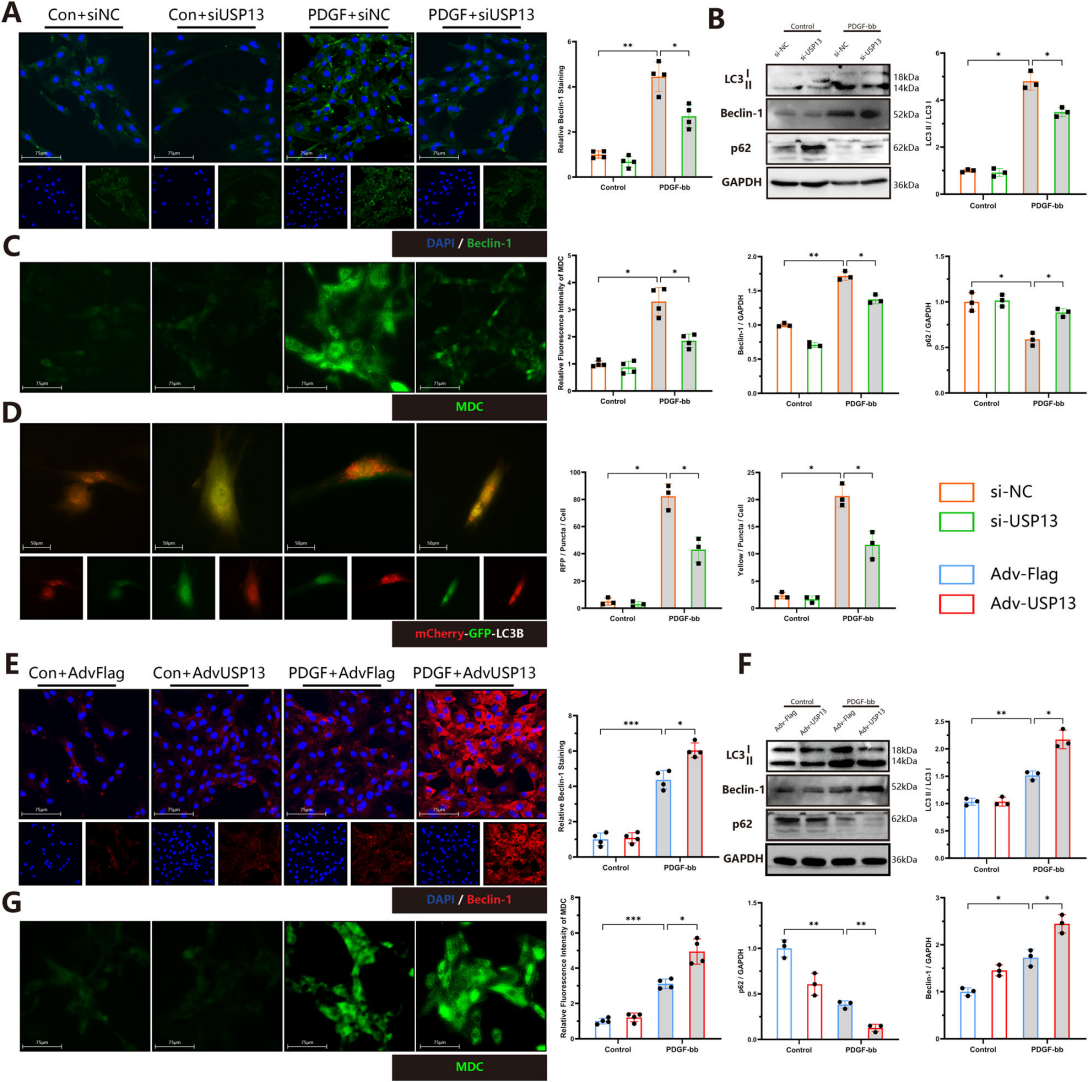
USP13 regulates the autophagy activity of VSMCs. **A** Immunostaining analysis of Beclin-1 (Green) and DAPI (Blue) accompanied by quantitative analysis (*n* = 4, scale bars = 75 μm). **B** Representative WB images and quantitative analysis of LC3, Beclin-1 and p62 (*n* = 3). **C** Representative images and quantitative analysis of MDC staining. (*n* = 4, scale bars = 75 μm). **D** mRFP-GFP-LC3 expressing rat SMCs were treated with si-NC or si-USP13 and then with or without PDGF-bb stimulation, *n* = 3, scale bars = 75 µm. **E** Immunostaining analysis of Beclin-1 (Red) and DAPI (Blue) accompanied by quantitative analysis (*n* = 4, scale bars = 75 μm). **F** Representative WB images and quantitative analysis of LC3, Beclin-1 and p62 (*n* = 3). **G** Representative images and quantitative analysis of MDC staining. (*n* = 4, scale bars = 75 μm). *p < 0.05, **p < 0.01, ***p < 0.001.

To further investigate the role of USP13 in Beclin-1-mediated autophagy, we assessed the impact of USP13 knockdown on autophagic activation induced by PDGF-bb. Knockdown of USP13 resulted in a significant inhibition of PDGF-bb induced autophagy, as evidenced by decreased levels of Beclin-1 and LC3 II, coupled with an increase in p62 (Fig. 7A, B). Additionally, MDC staining demonstrated reduced fluorescence intensity following USP13 knockdown, further supporting the inhibitory effect on autophagic activity (Fig. 7C). We also employed Adv-mCherry-GFP-LC3 infection to closely monitor changes in autophagic flux. As illustrated in the accompanying figures, USP13 knockdown markedly reduced the number of both yellow (indicating autophagosomes) and red puncta (indicating mature autolysosomes) in comparison to treatment with PDGF-bb alone, indicating a significant impairment in autophagic flux (Fig. 7D). Conversely, overexpression of USP13 promoted the activation of PDGF-bb-induced autophagy VSMCs, as evidenced by increased levels of Beclin-1 and LC3 II, a decrease in p62 (Fig. 7E, F), and enhanced MDC fluorescence intensity (Fig. 7G). These results collectively indicate that USP13 is a critical regulator of autophagy, modulating the autophagic response in VSMCs in the context of PDGF-bb stimulation.

### BHLHE40 transcriptionally activates USP13 in phenotypic transition of VSMCs

**Fig. 8:**
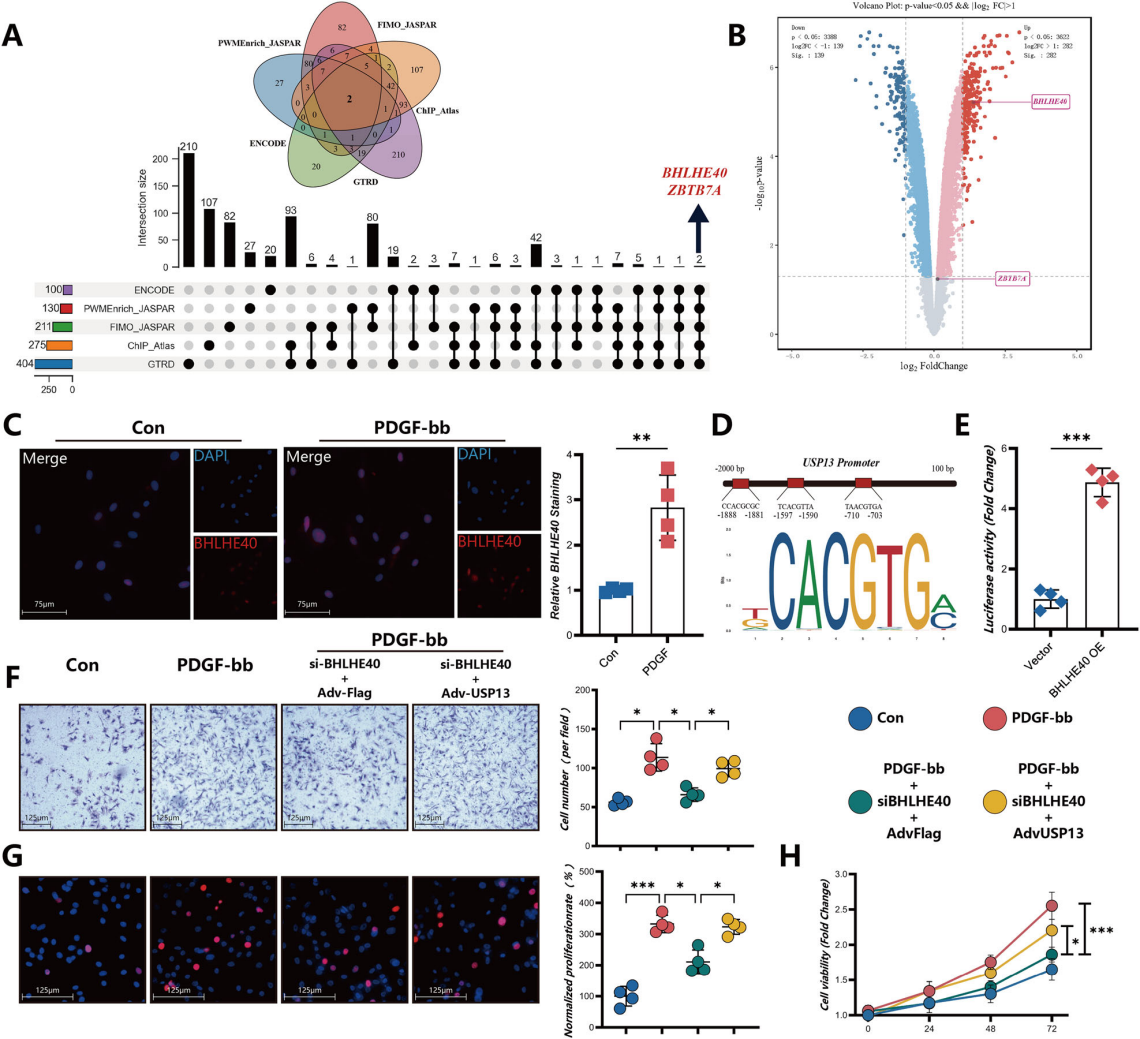
USP13 regulates the autophagy activity of VSMCs. **A** Upset plot of Venn diagram of potential transcription factors of USP13 predicted by databases. **B** Volcano plot showing the differential gene expression profiles in smooth muscle cells following PDGF-bb stimulation, derived from transcriptome analysis (GSE52488). **C** Immunostaining analysis of BHLHE40 (Red) and DAPI (Blue) accompanied by quantitative analysis (*n* = 4, scale bars = 75 μm). **D** JASPAR Predicted Binding Sites for BHLHE40. **E** The relative luciferase activity from vector plasmid or the BHLHE40 overexpression plasmid transfected 293t cells was measured by a dual luciferase reporter system. *n* = 4. **F** Representative images and quantitative analysis of the Transwell assay for VSMCs (*n* = 4, scale bars = 125 μm). **G** Representative EdU staining images and corresponding quantitative analysis (*n* = 4, scale bars = 125 μm). **H** The CCK-8 method was performed to detect the cell viability of VSMCs (*n* = 3). *p < 0.05, **p < 0.01, ***p < 0.001.

To elucidate the regulatory mechanism of USP13 in VSMCs, ENCODE, PWMenrich_JASPAR, FIMO_JASPAR, ChIP_Atlas, and GTRD were used to investigate the transcription factors that regulate USP13 (Fig. 8A). This analysis revealed two specific TFs, BHLHE40 and ZBTB7A, that bind to the promoter region of USP13. Subsequent examination of the GSE52488 dataset indicated a significant increase in BHLHE40 expression following PDGF-bb stimulation of SMCs, while the expression levels of ZBTB7A remained relatively unchanged (Fig. 8B). Immunofluorescence assays further validated this observation, demonstrating a notable increase in BHLHE40 fluorescence intensity upon PDGF-bb treatment (Fig. 8C). Luciferase reporter assays confirmed that overexpression of BHLHE40 correlates with enhanced transcriptional activity of the USP13 promoter, supporting the hypothesis that BHLHE40 is a key upstream regulator of USP13 (Fig. 8D). Importantly, silencing BHLHE40 resulted in a significant reduction in PDGF-bb-induced proliferation and migration of VSMCs (Fig. 8F–H). Co-overexpression of USP13 in BHLHE40-knockdown cells partially counteracted the decline in these cellular functions, suggesting that BHLHE40 influences VSMCs behavior primarily through its regulatory effects on USP13.

### Beclin-1 knockdown abolishes the aggravating effects of USP13 in VSMCs differentiation

**Fig. 9:**
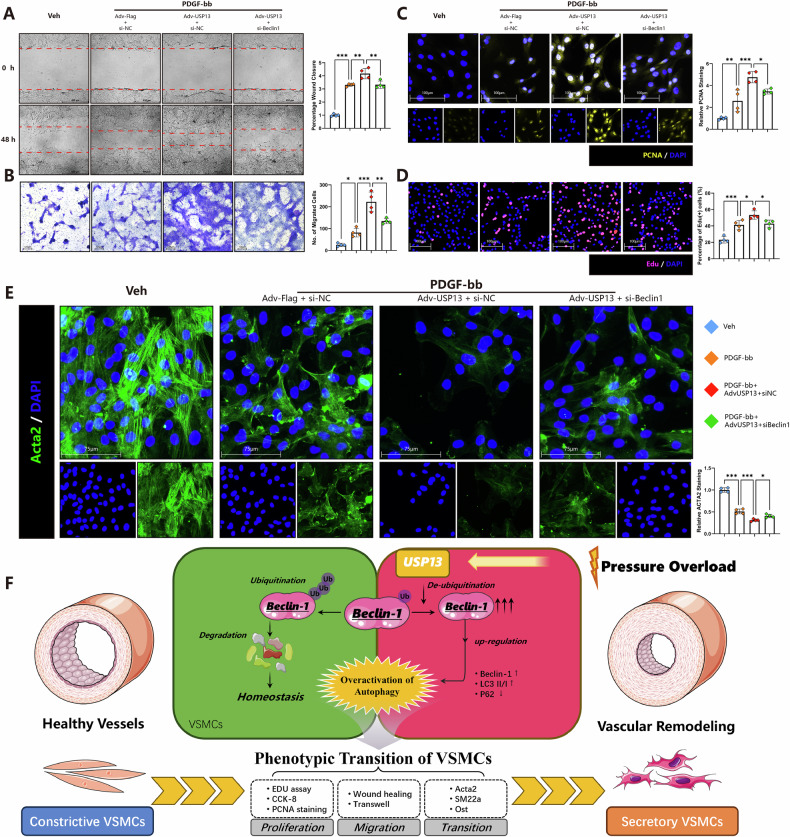
Beclin-1 knockdown abolishes the aggravating effects of USP13 in VSMCs differentiation. **A** Representative images and quantitative analysis of the wound healing assay for VSMCs (*n* = 4, scale bars = 650 μm). **B** Representative images and quantitative analysis of the Transwell assay for VSMCs (*n* = 4, scale bars = 650 μm). **C** Immunostaining of PCNA (Yellow) and DAPI (DAPI) accompanied by quantitative analysis (*n* = 4, scale bars = 125 μm). **D** Representative EdU staining images and corresponding quantitative analysis (*n* = 4, scale bars = 275 μm). **E** Immunostaining of Acta2 (Green) and DAPI (DAPI) accompanied by quantitative analysis (*n* = 4, scale bars = 125 μm). **F** Schematic diagram illustrating the molecular mechanisms underlying the protective effect of USP13 in vascular remodeling and phenotypic transition of VSMCs. *p < 0.05, **p < 0.01, ***p < 0.001.

To investigate whether the detrimental effects of USP13 are mediated through Beclin-1, we performed Beclin-1 knockdown in VSMCs utilizing siRNA. The scratch wound healing and Transwell migration assays demonstrated that Beclin-1 depletion significantly attenuated the enhanced VSMCs migratory ability induced by USP13 overexpression (Fig. 9A, B). Similarly, both EdU staining and PCNA immunostaining revealed that the knockdown of Beclin-1 markedly alleviated the USP13-induced increase in VSMCs proliferation (Fig. 9C, D). Furthermore, immunofluorescence analysis of the VSMCs contractile marker Acta2 demonstrated that Beclin-1 depletion mitigated the USP13-mediated suppression of Acta2 expression (Fig. 9E). Overall, these findings suggest that the pro-differentiation, promigratory, and proliferative effects of USP13 in VSMCs are significantly weakened upon Beclin-1 knockdown, highlighting the critical role of Beclin-1 in USP13-drivenVSMCs transition.

## Discussion

Pressure overload-induced vascular remodeling is a multifaceted pathophysiological phenomenon that plays a crucial role in the progression of various cardiovascular diseases [2, 3]. This process is primarily driven by increased mechanical stress on the vascular wall, which initiates a cascade of maladaptive responses in VSMCs [7, 23]. Initially, compensatory mechanisms such as VSMC hypertrophy and hyperplasia may occur; however, these changes ultimately lead to alterations in the extracellular matrix composition, enhanced vascular fibrosis, and a shift in fibroblast phenotype [5]. Finally, these adaptations contribute to persistent vascular stiffness and luminal narrowing [4]. Importantly, mechanical stretching can stimulate the production and release of PDGF-BB, increating a paracrine and autocrine loop that amplifies the synthetic VSMC phenotype. This establishes PDGF-BB as a crucial mechanistic link between mechanical stress and the subsequent cellular changes that define vascular remodeling. Recent investigations into vascular remodeling have increasingly focused on the molecular and cellular mechanisms driving the phenotypic transitions of VSMCs, with a particular emphasis on programmed cell death signaling pathways, especially autophagy [9, 11, 24]. In the current study, we present novel findings highlighting the role of USP13 in vascular remodeling and VSMC dynamics. Notably, we observed a substantial upregulation of USP13 protein levels during vascular remodeling induced by TAC. Additionally, PDGF-BB was found to induce a time-dependent increase in USP13 expression in both rat VSMCs and human aortic VSMCs. In vivo experiments demonstrated that USP13 exacerbates vascular wall thickening accompanying pressure overload, while concurrently downregulating the contractile protein Acta2. In vitro studies further revealed that USP13 promotes VSMC proliferation and migration, thereby exacerbating the phenotypic transition associated with pathological remodeling. Mechanistically, USP13 enhances autophagy through its deubiquitinating activity on Beclin-11. Collectively, these findings underscore the critical role of USP13 in mediating vascular responses to pressure overload and position it as a promising therapeutic target for addressing vascular remodeling disorders.

Vascular smooth muscle cells are integral components of the vascular wall, located within the medial layer of vessels [4]. Unlike cardiac or skeletal muscle cells, they exhibit a high degree of phenotypic plasticity, enabling them to adapt to various physiological and pathological stimuli [25, 26]. Under normal physiological conditions, VSMCs predominantly display a contractile phenotype, characterized by the expression of contractile proteins such as Acta2, Transgelin and myosin heavy chain, which are essential for maintaining vascular tone and integrity [6, 25]. However, in response to vascular injury or pathological stimuli, VSMCs can switch to a different phenotype [7], such as synthetic, osteo/chondrogenic and macrophage-like etc. The synthetic phenotype is marked by reduced contractile protein expression and increased secretion of extracellular matrix components and cytokines, facilitating tissue repair but also contributing to pathological processes like atherosclerosis and restenosis [27]. The osteo/chondrogenic phenotype is associated with vascular calcification and the macrophage-like phenotype may play a role in inflammatory responses [28]. Each of these phenotypic states implicates VSMCs in various cardiovascular diseases [7, 29], underscoring their versatility and complex role in vascular biology as well as highlighting they may serve as an exciting therapeutic target for cardiovascular disease. Notably, under conditions of pressure overload, underwent a shift from a contractile phenotype to a synthetic phenotype. This phenotypic shift is accompanied by a marked increase in their proliferative and migratory capacities, facilitating vascular remodeling and neointimal formation, which are key events in the pathogenesis of occlusive vascular diseases [3]. In this research, we illustrated USP13 significantly exacerbates vascular wall thickening induced by transverse aortic constriction. Furthermore, our findings demonstrated that USP13 enhances the proliferative and migratory capacities of VSMCs in response to PDGF-BB stimulation. This is also evidenced by the upregulation of synthetic markers and concomitant downregulation of contractile markers. Overall, our results reveal that USP13 plays a pivotal role in promoting vascular remodeling and facilitating the phenotypic transition of VSMCs.

Ubiquitin-specific protease 13, a pivotal member of the ubiquitin-specific protease family, plays a crucial role in numerous cellular processes through its deubiquitinating activity [30]. The USP family, characterized by its ability to cleave ubiquitin moieties from target proteins, thus regulates protein stability and function. The ubiquitination process serves as a counterbalance to deubiquitination, primarily by tagging proteins for degradation via the proteasome pathway [31]. This process is essential for various cellular pathways, influencing protein degradation, signal transduction, and the maintenance of cellular homeostasis. In our previous investigations, we explored the role and mechanisms of various E3 ubiquitin ligases, including WWP1 [32], TRIM35 [33] and Triad3A [34] etc., in cardiovascular diseases. Our findings indicated that WWP1, a ubiquitin E3 ligase, mitigates myocardial infarction by inhibiting the ubiquitination of KLF15, thereby reducing myocardial inflammation [32]. Furthermore, TRIM35 was found to interact with and ubiquitinate SLC7A5, subsequently upregulating the mTORC1 signaling pathway, playing a significant role in cardiac remodeling in pressure overload [33]. Additionally, Pellino1 was to be activated in vivo within pressure-overloaded rat hearts and in vitro in neonatal rat cardiac fibroblasts subjected to mechanical stretch [35]. Targeting Pellino1 provided therapeutic benefits by reducing cardiac fibroblast activation and cardiac fibrosis in response to pressure overload [35]. Within the area of cardiovascular research, members of the ubiquitin-proteasome system (UPS) also have garnered attention for their involvement in pathophysiological processes such as cardiac hypertrophy, heart failure, and vascular remodeling. Recent evidence has highlighted the significance of specific USP family members in vascular smooth muscle cell biology and vascular remodeling processes. USP15 and USP20 have attracted particular attention due to their potential roles in regulating key signaling pathways involved in VSMC phenotypic modulation. While the precise mechanisms through which these USPs influence vascular remodeling are still being elucidated, current research suggests they may function through multiple interconnected pathways including modulation of oxidative stress, regulation of inflammatory responses, and control of metabolic reprogramming in VSMCs. For example, USP20 associates with several components of the tnfr1 thus inhibits TNF-induced VSMCs inflammation and attenuates atherosclerosis [36]. Furthermore Wu et al. have illustrated that USP15 knockdown inhibited pulmonary artery VSMCs proliferation, migration, and YAP1/TAZ signaling in hypoxic pulmonary hypertension [37]. However, the specific role and underlying mechanisms of USP13 in the cardiovascular system remain unclear, particularly regarding pressure overload-induced vascular remodeling and the differentiation of VSMCs. In this study, we have elucidated that USP13 exacerbates vascular remodeling by enhancing the proliferation and migration of VSMCs. Importantly, we identified a novel interaction between USP13 and Beclin-1, where USP13 facilitates the deubiquitination of Beclin-1, thereby promoting autophagy. This interaction provides an insight into how USP13 modulates vascular remodeling, highlighting its potential as a target for therapeutic intervention in vascular disorders and VSMCs dysregulation.

Autophagy is a highly regulated programmed cell death process involving the degradation and recycling of cytoplasmic components through the lysosomal pathway [8]. Key molecules orchestrating this process include Beclin-11, which plays a pivotal role in the initiation of autophagosome formation, LC3, and p62/SQSTM1 [38]. Physiologically, autophagy plays a crucial role in maintaining cellular homeostasis by removing damaged organelles and proteins. However, its dysregulation has pathophysiological implications, particularly in cardiovascular diseases (CVDs) [39, 40]. Interestingly, autophagy serves as a double-edged sword in the context of CVDs. For example, activating autophagy inhibits NLRP3-mediated VSMCs pyroptosis in chronic kidney disease induced vascular calcification [41]. It is worth noting that overactivated autophagic flow is also recognized to exacerbate pressure overload-induced vascular remodeling [11, 22]. Inhibition autophagy by activating the transcription of miR-30a and that miR-30a-mediated autophagy defects could inhibit intimal hyperplasia in a carotid arterial injury model [22]. Nicotine-induced autophagy promotes the phenotype switching of VSMCs and accelerates atherosclerosis [42]. This underscores the complex relationship between autophagy and vascular health, suggesting that targeted modulation of autophagic pathways may hold therapeutic potential for mitigating vascular remodeling and maintaining vascular homeostasis. Beclin-1 is widely recognized as a crucial mediator of autophagy. Previous research has shown that dysregulated autophagy and Beclin-1 can led to excessive proliferation, migration, and differentiation of VSMCs, contributing to various vascular diseases [11, 22, 40]. Approaches to modulating autophagy, such as utilizing miRNA30a, have been explored for the treatment of vascular remodeling diseases. However, there is a scarcity of studies focusing on ubiquitination modifications to treat vascular remodeling. To address this gap, we have identified that targeting the USP13 effectively reduces the protein levels of Beclin-1. The inhibition of USP13 in vivo has been shown to alleviate pressure-induced arterial wall thickening and VSMC phenotypic transition, suggesting a potential therapeutic strategy for patients with clinical hypertension.

In summary, our study demonstrates that USP13 plays a pivotal role in the regulation of Beclin-1 deubiquitination and acts as a facilitator of vascular remodeling. Silencing USP13 expression effectively blocked PDGF-BB induced phenotype transition of VSMCs in vitro and stretch-induced vascular remodeling in vivo. These findings suggest a novel therapeutic strategy that could potentially mitigate the progression of hypertension and vascular diseases.

## Conclusion

In summary, this study elucidates the role of USP13 in the context of pressure overload-induced vascular remodeling, a critical process linked to cardiovascular diseases. By utilizing TAC murine model and PDGF-bb cellular model, we demonstrated that the levels of USP13 levels in vascular remodeling and phenotypic transition of VSMCs. Overexpression of USP13 exacerbates vascular wall thickening in response to TAC, while inhibition USP13 with Spautin-1 offers protective effects. Functionally, USP13 promotes VSMC proliferation and migration, as reflected in diminished PCNA levels and enhanced EdU incorporation following PDGF-BB treatment. Mechanistically, USP13 is shown to interact with and deubiquitinate Beclin-1, thus facilitating autophagic flux and contributing to VSMC phenotypic transition. These insights reveal USP13 as a pivotal modulator in VSMCs dynamics and highlight its potential as a therapeutic target for preventing pathological remodeling in cardiovascular diseases.

## Materials and methods

### Animal experiments

All animals were housed in a specific pathogen-free (SPF) facility under a controlled 12-h light/dark cycle. The experimental procedures involving animals were conducted in accordance with the Guide for the Care and Use of Laboratory Animals and received approval from the Institutional Animal Care and Ethics Committee at Xiamen University. Wild-type male mice of the C57BL/6 strain, spontaneously hypertensive rats (SHRs) and Wistar-Kyoto rats (WKYs) were obtained from Gempharmatech Co., Ltd., and were provided with a commercial diet and water available ad libitum.

To achieve the overexpression of USP13 in vivo, we utilized adeno-associated virus serotype 9 (AAV9). Specifically, we administered a total of 1.0 × 10^11^ vg/g of AAV9-USP13 via tail vein injection and the control group received an equivalent dose of empty vector virus. Following a 3-week period for viral expression, we proceeded with the surgical model to induce vascular remodeling. To inhibit USP13 activity in vivo, we employed the USP13 inhibitor, Spautin-1, for our experimental protocol. Spautin-1 was administered at a dosage of 20 mg/kg, delivered via intraperitoneal injection every 2 days post-surgery. This treatment regimen continued for 4 weeks, after which we collected vascular samples for further analysis.

### Artery remodeling model

Arterial remodeling induced by pressure overload was achieved through transverse aortic constriction as described previously methods [33, 43, 44]. Eight-week-old mice were anesthetized with 4% isoflurane and maintained on 1% isoflurane throughout the procedure. Following a thoracotomy to expose the aorta, a 5-0 silk suture, placed over a 27 G needle, was inserted between the innominate and left carotid arteries after the transverse thoracic aorta was clearly identified. The silk suture was then tightened quickly, and the needle was promptly removed to ensure consistent constriction. In contrast, the sham group underwent identical preparation without any aortic ligation. Post-surgery, the mice were placed on a heating pad and closely monitored until they regained full consciousness.

### Carotid artery ultrasound imaging

For carotid artery ultrasound imaging in mice, we employed the Vevo 2100 ultrasound system. In summary, the mouse was anesthetized using isoflurane, and hair in the neck region was carefully removed. Doppler mode was applied to detect carotid artery blood flow, while M-mode ultrasonography was utilized to assess luminal diameter and vascular wall thickness.

### Frozen section

The isolated carotid arteries were fixed with 4% paraformaldehyde for 24 h. After dehydration, the vascular samples were carefully exercised and immediately embedded in optimal cutting temperature (OCT). The embedded tissues were then snap-frozen in liquid nitrogen to maintain their structural fidelity. Using a cryostat set to −20 °C, sections were precisely cut into 6–8 micrometer thick slices. These sections were then transferred to positively charged glass slides, air-dried briefly, and stored at −80 °C until subjected to histological or immunohistochemical analyses.

### Hematoxylin-eosin (HE) staining

Hematoxylin-eosin (HE) staining was conducted according to routine protocols by using HE-staining kit. The frozen vascular sections were warmed to room temperature before being fixed in 10% neutral-buffered formalin for 30 min. Following fixation, the sections were rinsed in distilled water and sequentially stained with hematoxylin for 15 min. After differentiation in 1% acid alcohol, the slides were exposed to bluing in a mildly alkaline solution. The eosin staining was carried out for 1 min to impart pink. Finally, slides were dehydrated through ascending grades of ethanol, cleared in xylene, and mounted with a coverslip.

### Immunohistochemical and immunofluorescence staining

Immunohistochemical and immunofluorescence staining were used to detect the variation in protein levels. For the immunofluorescence staining process, the frozen sections were initially blocked using 3% bovine serum albumin (BSA) for 30 min at room temperature to prevent nonspecific binding. The sections were then incubated overnight with the specified primary antibodies. On the following day, the sections were washed three times with PBS and subsequently incubated with fluorescent-labeled secondary antibodies for 30 min at room temperature. DAPI was included to counterstain and visualize the nuclei. As for the immunohistochemical staining, sections were first treated with 3% hydrogen peroxide solution for 25 min to quench endogenous peroxidase activity. This was followed by a blocking step with 3% BSA for 30 min at room temperature, and the sections were subsequently incubated overnight with the desired antibodies in a diluent buffer. After washing three times with PBS, the sections were exposed to an AEC chromogen solution to develop the color reaction.

### Cell culture and treatment

Primary vascular smooth muscle cells were isolated from the aortas of Sprague-Dawley (SD) wild-type rats following established protocols. In brief, rats were humanely euthanized via cervical dislocation, and the aortas were aseptically excised and sectioned into pieces approximately 1 mm² in size. These tissue fragments were placed in T25 cell culture flasks and allowed to adhere for 4–6 h. Subsequently, a culture medium consisting of DMEM/F12 supplemented with 20% fetal bovine serum (FBS) and 1% penicillin-streptomycin was added. The medium was initially changed after 3 days and subsequently refreshed every 3 days. After about a month, when the cell confluence reached approximately 80%, the VSMCs were passaged. To simulate pressure overload induced VSMCs injury, we utilized the FlexCell Tension System (FlexCell International Corp.) to apply cyclic stretch to VSMCs cultured on collagen I–coated membranes. Cells were subjected to two stretch regimens: 5% elongation at 1 Hz to mimic physiological pulsatile conditions, and 18% elongation at 1 Hz to replicate pathological hypertensive stretch. Each condition was maintained for 24 h under standard culture conditions. For the experiments, VSMCs at the third to fourth passages were utilized. Additionally, human vascular smooth muscle cells were procured from ScienCell. To induce phenotypic transition, the VSMCs were serum-starved in DMEM/F12 without FBS for 24 h, followed by treatment with 20 ng/mL of platelet-derived growth factor-BB (PDGF-BB).

### Adenovirus‑mediated USP13 overexpression in vitro

To achieve overexpression of USP13 in vitro, the overexpression adenoviral were procured from GeneChem. When rat VSMCs reached approximately 80% confluence on culture plates, they were transitioned to a serum-free medium and transfected with either Adv-USP13 or Adv-Flag at a multiplicity of infection (MOI) of 50. After a 6-h incubation period, the adenoviral medium was replaced with DMEM/F12 supplemented with 10% fetal bovine serum (FBS). Subsequent experiments were performed 24-h post infection. The efficiency of USP13 overexpression was confirmed through Western blot analysis (Fig. S4).

### SiRNA and RNA interference

For the knockdown experiments, small interfering RNAs (siRNAs), specifically si-NC and si-USP13, were synthesized by RiboBio Corporation. The sequences for these siRNAs are detailed in Supplementary Fig. S3. VSMCs were seeded onto plates and allowed to adhere overnight. Transfection was performed using riboFECT™ CP, following the manufacturer’s protocol. Twenty-four hours post-transfection, the culture medium was refreshed, and further experimental procedures were carried out.

### Western blot assay

Protein expression levels were assessed through Western blot analysis. Initially, the samples were lysed using RIPA buffer containing protease and phosphatase inhibitor. The protein concentrations were quantified with a BCA protein assay kit. Subsequently, 20 μg of protein samples were separated by SDS-PAGE and transferred onto PVDF membranes. Following transfer, membranes were blocked with 5% non-fat milk dilution in TBST to prevent nonspecific binding and then incubated overnight at 4 °C with appropriate primary antibodies. The next day, membranes were washed three times by TBST and treated with HRP-conjugated secondary antibodies for an hour at room temperature. Protein bands were visualized using an enhanced chemiluminescence detection system, and images were captured with a chemiluminescence imaging system. Densitometric analysis of the bands was performed using ImageJ software to quantify protein expression levels.

### Co-immunoprecipitation and ubiquitination assay

To investigate protein-protein interactions, co-immunoprecipitation assay was performed. Cell lysates were prepared in NP40 IP lysis buffer containing a cocktail of protease inhibitors to preserve target proteins. The lysates were incubated overnight at 4 °C with specific antibodies conjugated to protein A/G Beads agarose beads. Post-incubation, the beads were extensively washed with cold IP wash buffer to remove non-specifically bound proteins. The immunoprecipitated complexes were eluted and then subjected to SDS-PAGE and Western blot analysis to assess protein-protein interaction and ubiquitination.

### RNA isolation and relative quantitative RT-PCR

Total RNA was extracted using Trizol reagent following the manufacturer’s protocol. After removal of genomic DNA, 500 ng RNA was transcribed into cDNA with Takara reverse transcription kit. mRNA levels of genes were quantitatively examined by RT-PCR using Fast SYBR™ Green Master Mix. The relative expression of genes was quantified by the 2 − ΔΔCt method. GAPDH served as the endogenous reference gene. The primer sequences are listed in Supplementary Table S1.

### Cell Counting Kit-8 (CCK8) assay

CCK-8 cell counting assay was used to detect the influence of USP13 on the proliferative capacity of VSMCs. As described in the previous literature, 1000 VSMCs were cultured in 96-well plates and received the relevant treatments. After adding 10 μl CCK8, the plates were incubated 37 °C for 2 h and the proliferative ability of the cells was measured at 450 nm.

### 5-Ethynyl-2’-deoxyuridine (Edu) staining

VSMCs proliferation ability was evaluated using the EdU (5-ethynyl-2’-deoxyuridine) incorporation assay. Cells were treated with different stimuli and subsequently incubated with 10 μM EdU for 2 h to ensure adequate incorporation into newly synthesized DNA. Following incubation, cells were fixed with 4% paraformaldehyde for 30 min at room temperature, permeabilized with 0.5% Triton X-100 for 20 min and then subjected to the EdU detection reaction as per the manufacturer’s protocol. A fluorescent azide was used to conjugate to EdU, allowing visualization of proliferating cells under a fluorescence microscope.

### Wound healing assay

The wound healing assay was employed to assess VSMCs migration capabilities. Cells were seeded into a 6-well plate and cultivated to near confluence. Subsequently, a sterile 200 μl pipette tip was used to create a linear scratch in the monolayer cell surface. VSMCs were then incubated with serum-free medium to ensure that the closure of the wound was due to cell migration rather than proliferation. Wound closure was monitored at 0, 24, and 48 h, and images were captured using a microscope. The wound area was quantified using ImageJ software, and the percentage of wound closure was calculated by comparing the initial wound area to the area at each time point.

### Transwell assay

Briefly, VSMCs were serum-starved overnight to synchronize their cell cycle, then harvested and resuspended in serum-free medium. A total of 200 ul serum-free medium containing 5 × 10^4^ cells were added into the upper chamber of transwell inserts with an 8 μm pore size (Corning), with the lower chamber filled with medium containing 10% FBS. After 24 h of incubation at 37 °C, non-migratory or non-invasive cells remaining in the upper chamber were gently removed with a cotton swab. Migrated cells on the underside of the membrane were fixed with methanol, stained with crystal violet, and counted in random fields under a light microscope.

### Measurement of fluorescent of LC3 puncta

To investigate the progression of autophagy, the mCherry-GFP-LC3B adenoviral system for the transduction of target VSMCs. Briefly, the mCherry-GFP-LC3B adenovirus was added to the culture medium. Following incubation for 24 h, the medium was replaced with fresh growth medium to eliminate excess viruses. Fluorescent images were captured using a confocal laser scanning microscope, allowing for the quantitative analysis of the mCherry and GFP signals.

### Monodansylcadaverine staining

Monodansylcadaverine (MDC) staining was performed to detect autophagy in cells, following the manufacturer’s instructions provided in the MDC Autophagy Detection Kit (Beyotime). Briefly, cells were cultured in plates and treated under the experimental conditions. After treatment, the cells were incubated with 0.05 mM MDC staining solution at 37 °C for 30 min in the dark. Subsequently, the cells were washed three times with PBS and fluorescence images were captured immediately using a fluorescence microscope with an emission wavelength of 525 nm.

### Dual luciferase reporter assay

HEK293T cells were transfected with the USP13-luc reporter, BHLHE40, and pRL-TK plasmids simultaneously. Following transfection, luciferase activity was measured using a dual luciferase reporter assay kit (Beyotime), in accordance with the manufacturer’s instructions.

### Statistical analysis

The results are presented as means ± standard deviation (SD). Statistical analyses were conducted using GraphPad Prism 9.5. An unpaired *t*-test was used to compare two groups. For comparisons involving more than two groups, one-way analysis of variance (ANOVA) was employed, followed by post hoc testing using Tukey’s method. A *p* value of less than 0.05 was considered indicative of statistical significance in all analyses performed.

## Supplementary information

Supplementary Materials

Uncutted Gel
