# Supplementary material for: USP13 facilitates pressure overload induced vascular remodeling and phenotypic transition of VSMCs via deubiquitinating Beclin-1
Source: Cell Death Discov. 2026 Jan 3;12:76. doi: 10.1038/s41420-025-02931-w (PMC12858841; doi:10.1038/s41420-025-02931-w)
Supplement: Supplementary file 1 — Supplementary Materials [file 41420_2025_2931_MOESM1_ESM.docx]

**USP13 Facilitates Pressure Overload Induced Vascular Remodeling and Phenotypic Transition of VSMCs Via Deubiquitinating Beclin-1**

**Rui-Qiang Qi^1, 4, †^, Qi-Fei Xie^1, 2, †^, Liu-Hang Su^1^, Yan Wang^1^,** **Sui-Ji Li ^1^*, Xia Lu^3^*, Juan Song^1^***

**^1^** Xiamen Cardiovascular Hospital of Xiamen University, Xiamen University, Xiamen, Fujian, 363001, China.

^2^ Department of Nuclear Medicine, the Third Affiliated Hospital of Soochow University, Changzhou, Jiangsu Province, China

^3^ Department of Cardiology, Shanghai Sixth People’s Hospital Affiliated to Shanghai Jiao Tong University School of Medicine, Shanghai 200233, China.

**^4^** Heart Center and Beijing Key Laboratory of Hypertension, Beijing Chaoyang Hospital, Capital Medical University. Beijing 100020, China.

***Corresponding Author:** Juan Song. Ph.D. Email: songjuan_ok@163.com; Xia Lu, Ph. D. Email: xialu0292@163.com; Sui-Ji Li, Ph. D. Email: drlisuiji@163.com.

**SUPPLEMENTAL MATERIALS**

**Supplementary Table**

**Supplementary Table S1. Sequences of primers (5'->3') used for qRT-PCR.**

| **Species** | **Gene** | **Sequence** | |
| --- | --- | --- | --- |
| **Rat** | **Usp13** | forward | ACTCACCATTAGATCCAACGC |
|  |  | reverse | CTCCTCCTTCATCACCTGTTC |
| **Human** | **Usp13** | forward | TCTCCTACGACTCTCCCAATTC |
|  |  | reverse | CAGACGCCCCTCTTACCTTCT |

**Supplementary Table S2**

**Antibodies used in Western blots.**

| **Antibody** | **Manufacturer** | **Product**  **number** | **Host** | **Dilution** |
| --- | --- | --- | --- | --- |
| USP13 | Proteintech | 16840-1-AP | Rabbit | 1: 2000 |
| Beclin-1 | Proteintech | 66665-1-Ig | Mouse | 1: 5000 |
| Osteopontin | Proteintech | 22952-1-AP | Mouse | 1: 2000 |
| Transgelin | Proteintech | 10205-2-AP | Rabbit | 1: 2000 |
| ACTA2 | Boster | BM0002 | Mouse | 1: 1000 |
| LC3B | Abclonal | A7198 | Rabbit | 1: 1000 |
| P62 | Abclonal | A21702 | Rabbit | 1: 1000 |
| GAPDH | Abcam | ab8245 | Rabbit | 1: 2500 |
| Ubiquitin (K48) | Abcam | EP8589 | Rabbit | 1: 5000 |

**Antibodies used in Immunofluorescence and Immunohistochemistry.**

| **Antibody** | **Manufacturer** | **Product**  **number** | **Host** | **Dilution** |
| --- | --- | --- | --- | --- |
| USP13 | Proteintech | 16840-1-AP | Rabbit | 1: 400 |
| Beclin-1 | Proteintech | 66665-1-Ig | Mouse | 1: 200 |
| Osteopontin | Abclonal | A21084 | Mouse | 1: 100 |
| PCNA | Proteintech | 10205-2-AP | Rabbit | 1: 400 |
| ACTA2 | Boster | BM0002 | Mouse | 1: 400 |
| BHLHE40 | Proteintech | 17895-1-AP | Rabbit | 1: 200 |


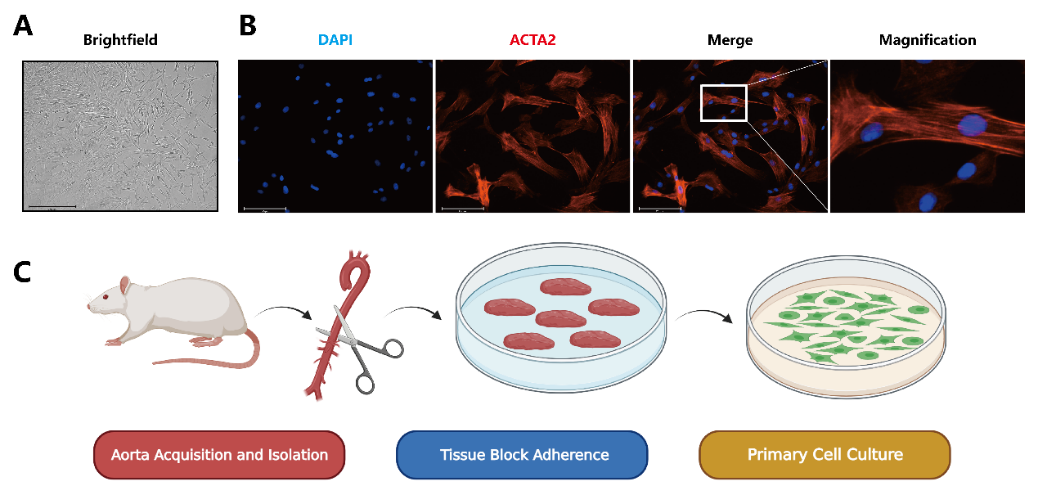
**Supplementary Figures and** **Figure Legends**

**Supplementary Figure S2. Isolation and characterization of primary rat aortic smooth muscle cells (SMCs).** Wild-type rats were euthanized, and aortas were harvested under sterile conditions for primary culture. **(A)** A microscopic image of the isolated VSMCs. **(B)** Immunofluorescence staining using the VSMCs marker Acta2 (Red) was employed to identify the VSMCs.


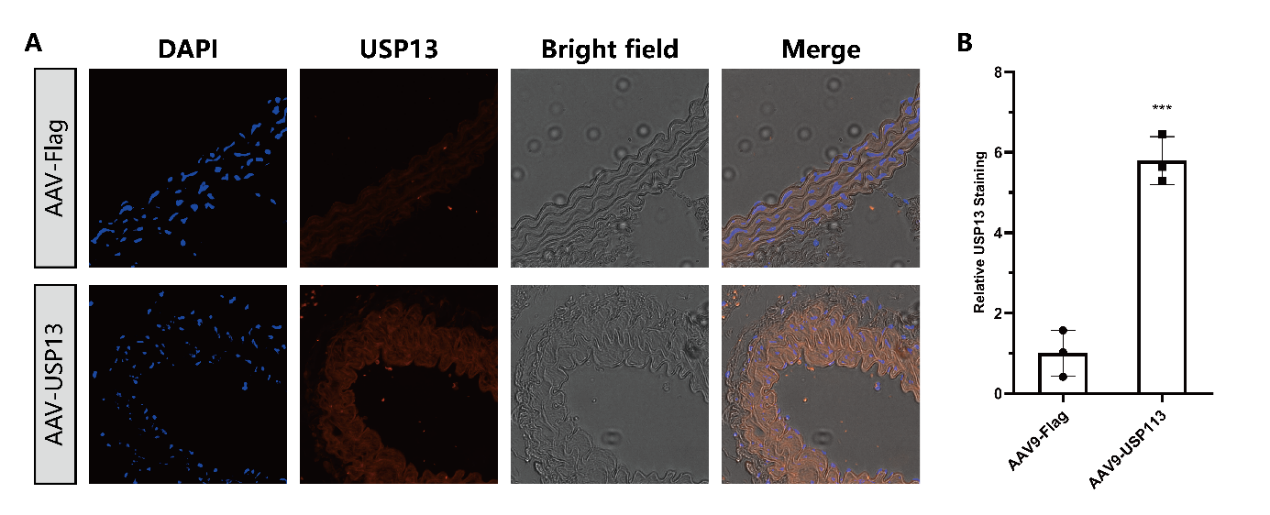


**Supplementary Figure S2.** **USP13 overexpression efficiency detection in vivo.** C57BL/6J mice were administered with a tail vein injection of 1.0 × 10^11 vg/g of AVV9-USP13 and an equivalent amount of AVV9-Flag. After a three-week period to allow for viral expression, the mice were euthanized, and arterial tissues were subsequently collected to assess the efficiency of USP13 overexpression. **(A)** Representative immunostaining images and **(B)** quantifications of USP13 (n=3 per group) . AAV9, Adeno-Associated Virus serotype 9; DAPI, 4',6-diamidino-2-phenylindole. **P < 0.01.


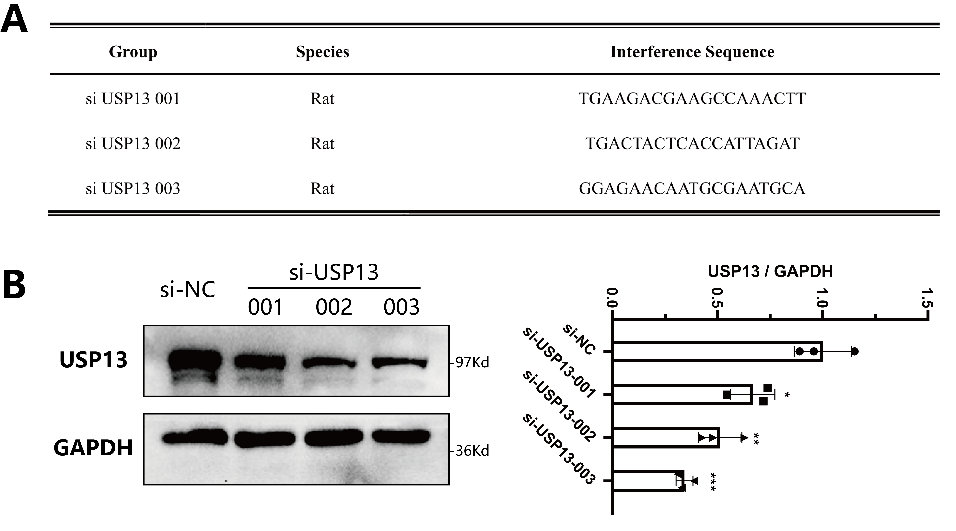


**Supplementary Figure S3. USP13 interference efficiency validation invitro.** Primary rat vascular smooth muscle cells were transfected and subsequently analyzed for the interference efficiency of USP13 after 48 hours. **(A)** The interference sequences of the USP13 small interfering RNA are detailed. **(B)** Representative images of Western blot analysis for USP13, along with corresponding statistical data (n=3 per group). *P < 0.05, **P < 0.01, ***P < 0.001.


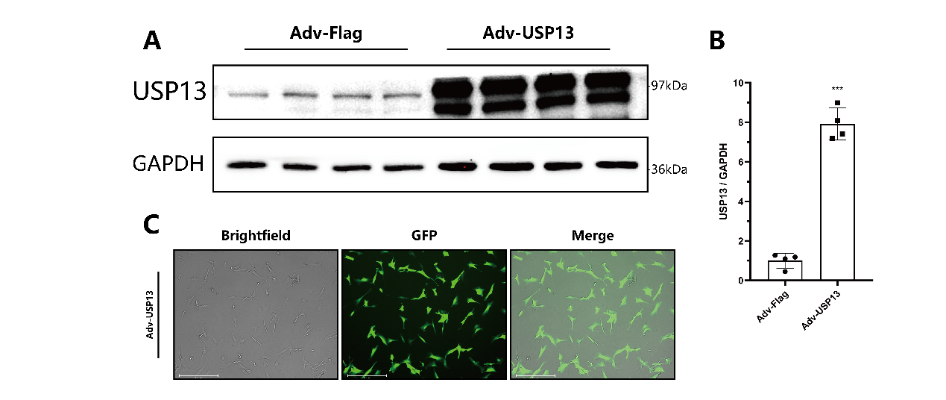


**Supplementary Figure S4. Validation of USP13 overexpression in-vitro.** After infecting primary rat VSMCs with an adenoviral vector for 48 hours, changes in USP13 protein levels were assessed. **(A)** Representative Western blot images and **(B)** statistical analysis of USP13 expression after infection. **(C)** Fluorescence microscopy was employed to evaluate the expression levels of the fluorescent protein. ***P < 0.001.

**
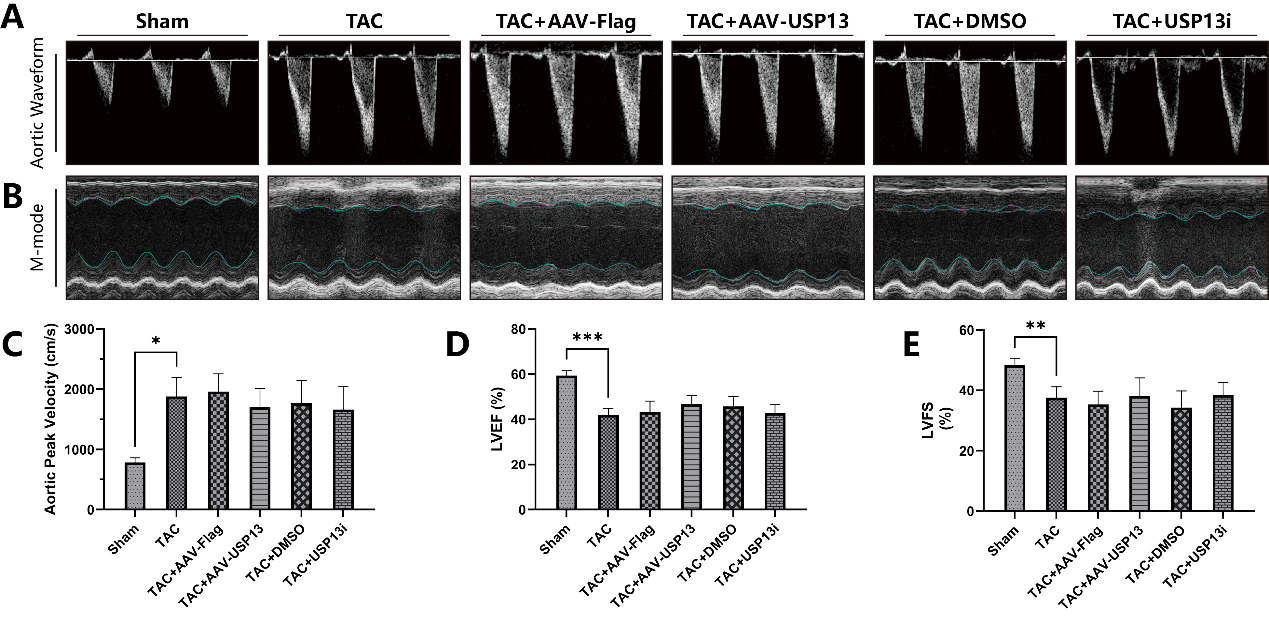
Supplementary Figure S5. Impact of USP13 on cardiac function. (A)** Representative image of aortic peak velocity at the site of aortic constriction. **(B)** Representative M-mode echocardiographic image of cardiac function. **(C)** Statistical analysis of blood flow velocity measurements at the site of aortic constriction in mice. N=5 per group. **(D-E)** Bar graphs showing the left ventricular ejection fraction (LVEF) and left ventricular fractional shortening (LVFS) as indicators of cardiac function. N=5 per group. *P < 0.05, **P < 0.01, ***P < 0.001.

**Supplementary Figure S7. USP13 maintains the contractility of vascular smooth muscle cells. (A-B)** Representative images and quantitative data from the gel contraction assay (n=5 per group). Scale bar=2mm.
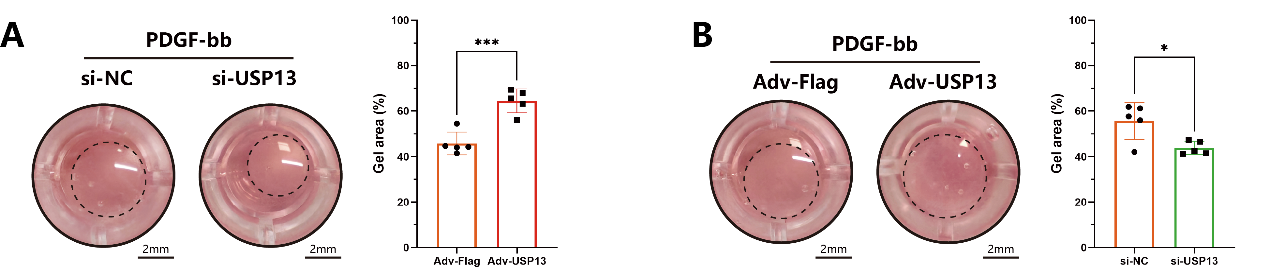
 *P < 0.05, ***P < 0.001.

**Supplementary Figure S8. USP13 mitigates PDGF-BB induced phenotypic transition of VSMCs in MOVAS. (A)** Representative EdU staining images and corresponding quantitative analysis (n=5 per group, scale bars=125 μm).
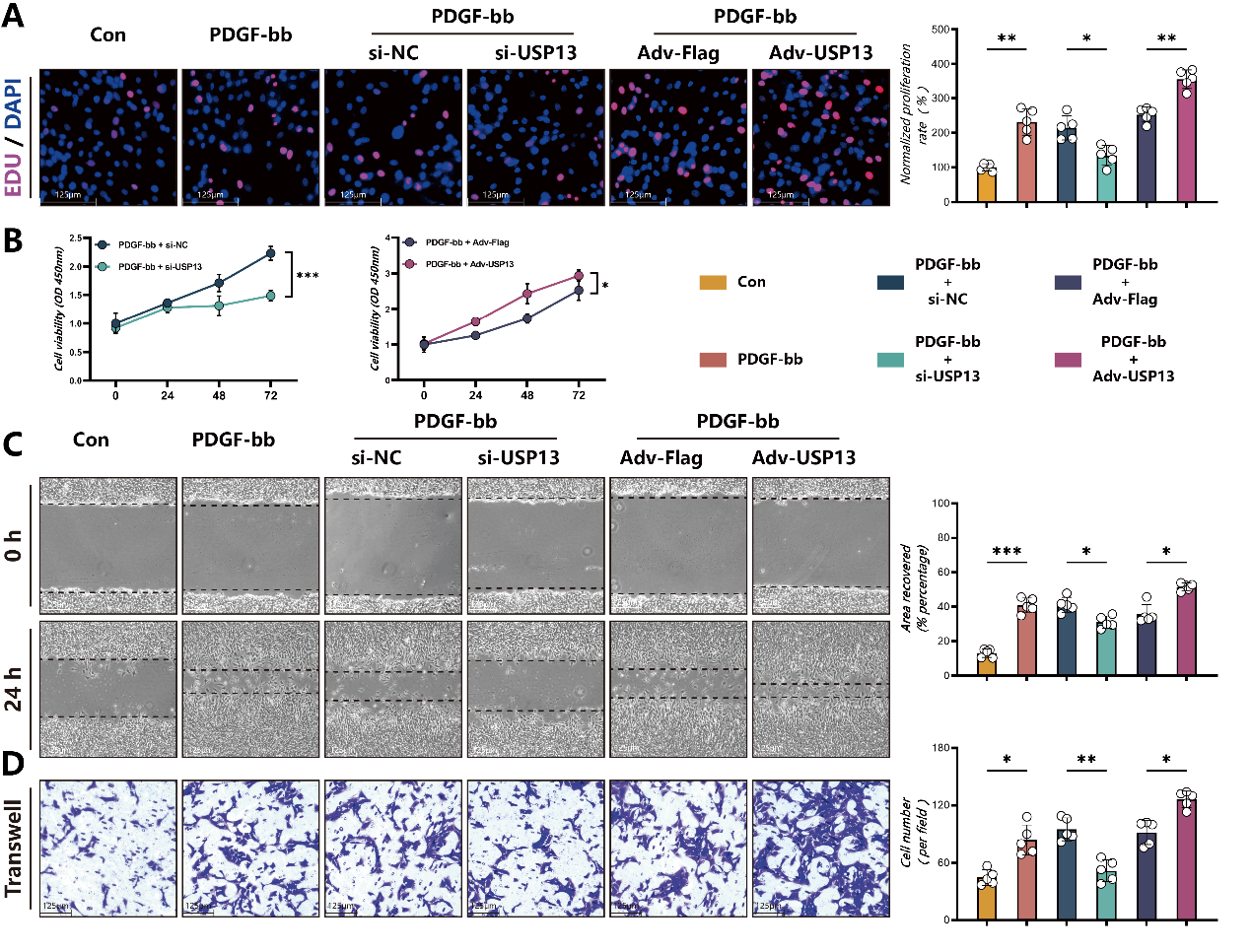
 **(B)** The CCK-8 method was performed to detect the cell viability of MOVAS (n=3 per group). **(C)** Representative images and quantitative analysis of the wound healing assay for VSMCs (n=5 per group, scale bars=125 μm). **(D)** Representative images and quantitative analysis of the Transwell assay for VSMCs (n=5 per group, scale bars=125 μm). * P < 0.05, ** P < 0.01, *** P < 0.001.
