# Supplementary material for: USP13 facilitates pressure overload induced vascular remodeling and phenotypic transition of VSMCs via deubiquitinating Beclin-1
Source: Cell Death Discov. 2026 Jan 3;12:76. doi: 10.1038/s41420-025-02931-w (PMC12858841; doi:10.1038/s41420-025-02931-w)

Fig1 D USP13

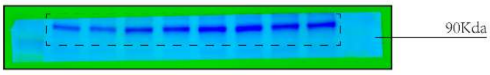

Fig1 D Acta2

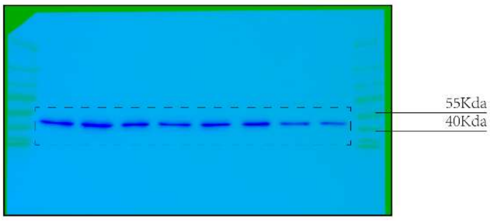

Fig1 D Osteopontin

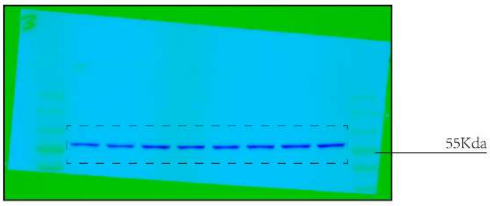

Fig1 D Transgelin

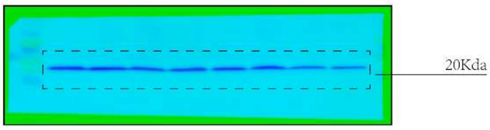

Fig1 D GAPDH

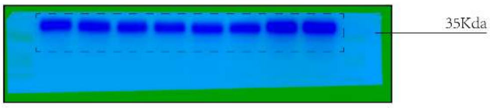

Fig1 G USP13

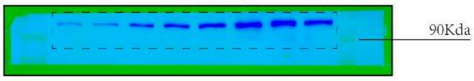

Fig1 G Acta2

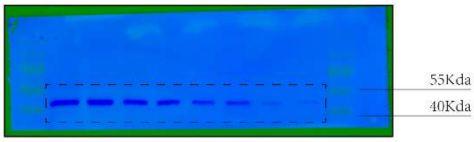

Fig1 G Osteopontin

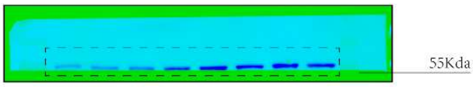

Fig1 G Transgelin

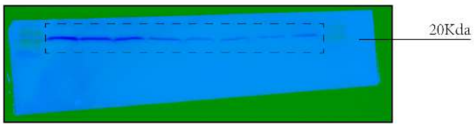

Fig1 G GAPDH

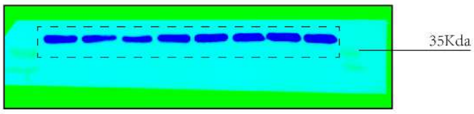

Fig2B USP13

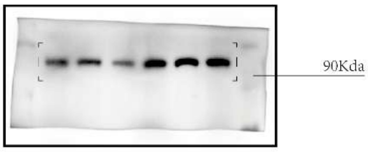

Fig2B GAPDH

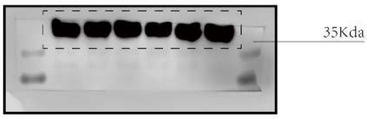

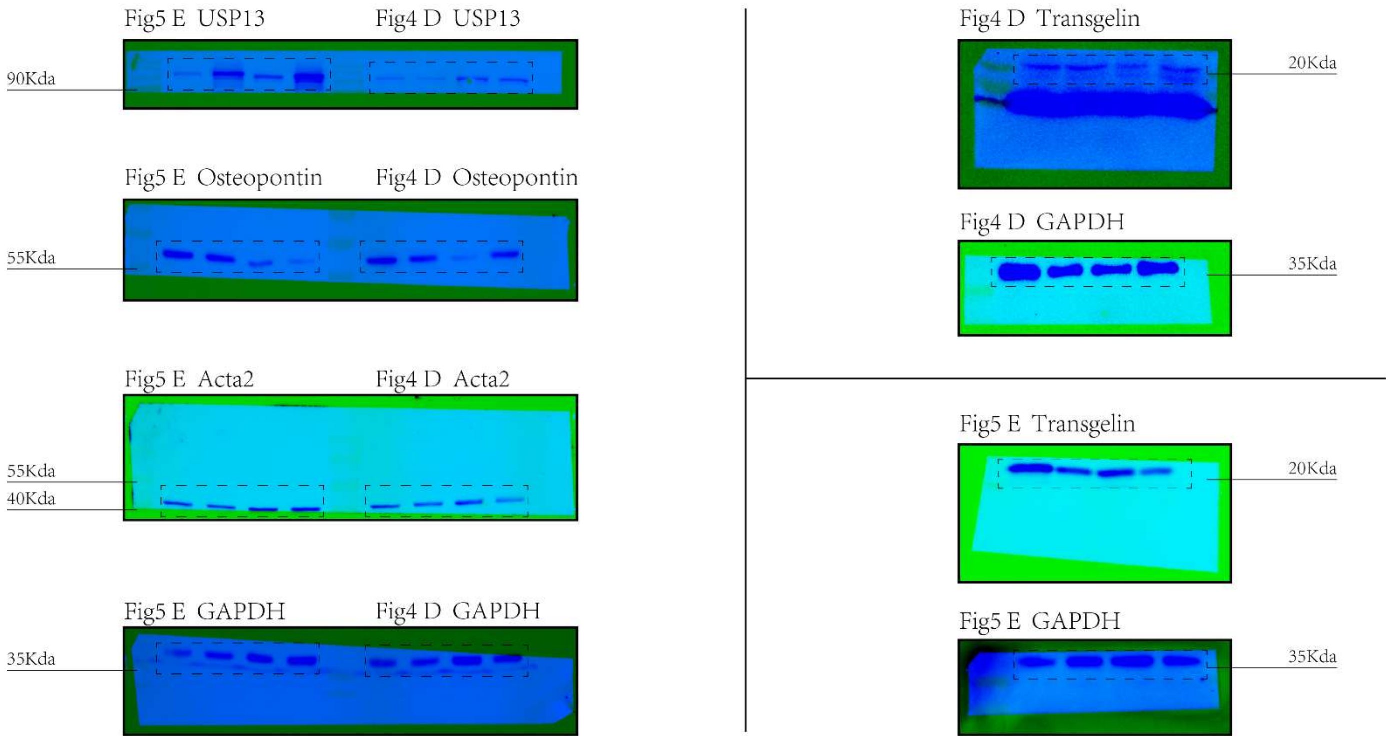
Fig5 E USP13
Fig4 D USP13
90Kda
Fig5 E Osteopontin
Fig4 D Osteopontin
55Kda
Fig5 E Acta2
Fig4 D Acta2
55Kda
40Kda
Fig5 E GAPDH
Fig4 D GAPDH
35Kda
Fig4 D Transgelin
20Kda
Fig4 D GAPDH
35Kda
Fig5 E Transgelin
20Kda
Fig5 E GAPDH
35Kda

Fig6 F MYC-USP13

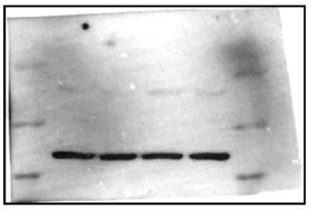

Fig6 F Flag-Beclin-1

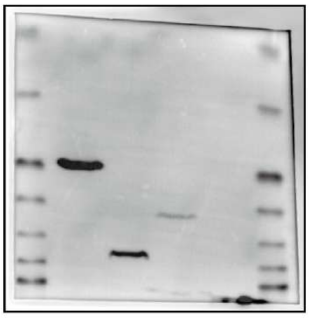

Fig6 F Flag-Beclin-1

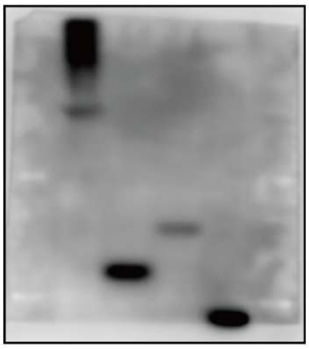

Fig6 G HA-UB

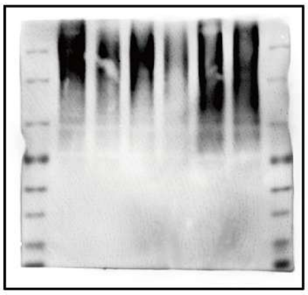

Fig6 G Flag-Beclin-1

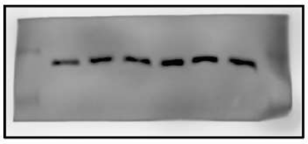

Fig6 G HA-UB

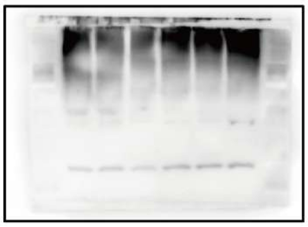

Fig6 G GAPDH

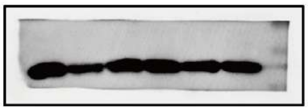

Fig6 H Ub

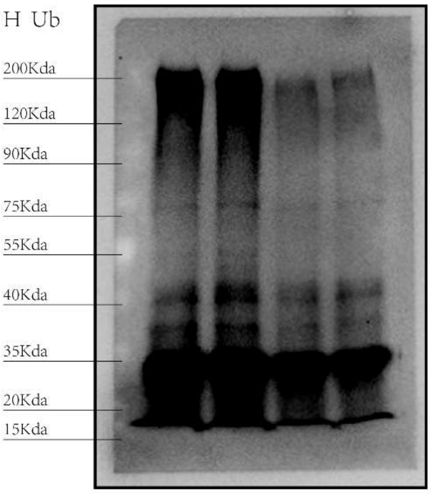

Fig6 H Beclin-1

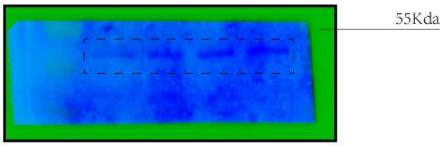

Fig6 H GAPDH

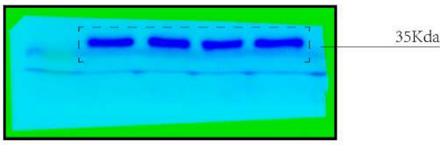

Fig6 I Ub

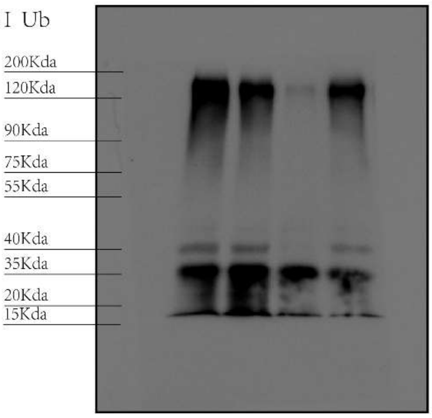

Fig6 I Beclin-1

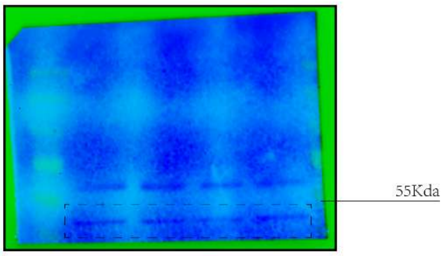

Fig6 I GAPDH

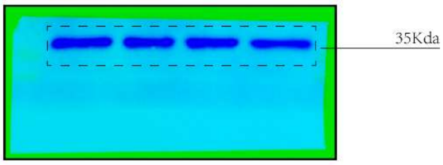

Fig6 J Ub

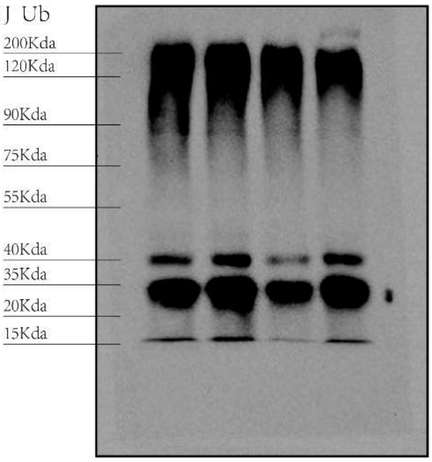

Fig6 J Beclin-1

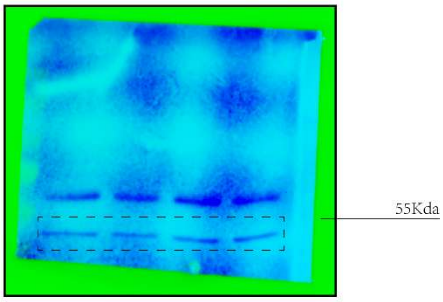

Fig6 J GAPDH

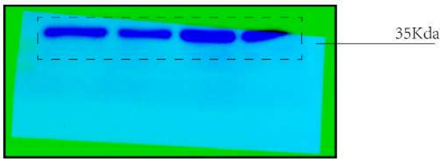

Fig7 B LC3

15Kda

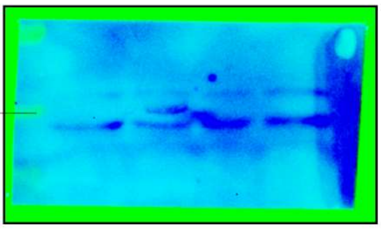

Fig7 B Beclin-1

55Kda

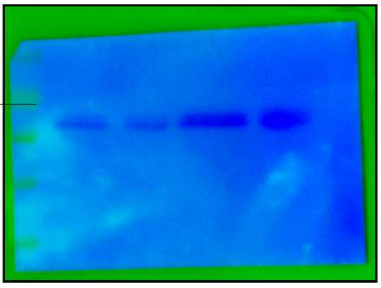

Fig7 B p62

75Kda

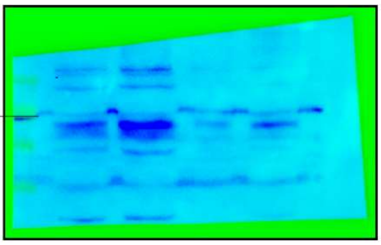

Fig7 B GAPDH

35Kda

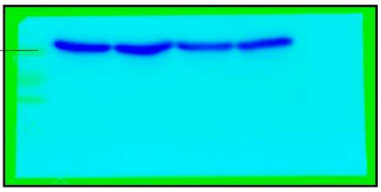

Fig7 F LC3

15Kda

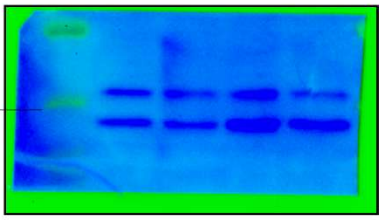

Fig7 F Beclin-1

55Kda

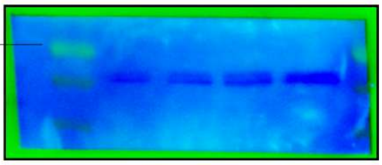

Fig7 F p62

75Kda

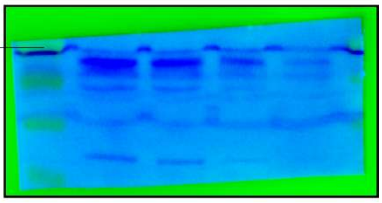

Fig7 F GAPDH

35Kda

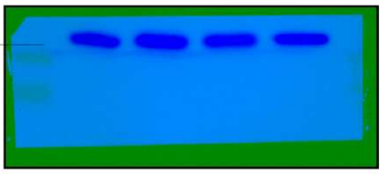

Supplement: Supplementary file 2 — Uncutted Gel [file 41420_2025_2931_MOESM2_ESM.pdf]
